# Evaluation of hydrogen production via steam reforming and partial oxidation of dimethyl ether using response surface methodology and artificial neural network

**DOI:** 10.1038/s41598-024-66402-5

**Published:** 2024-07-06

**Authors:** Karim Mansouri, Fatemeh Bahmanzadegan, Ahad Ghaemi

**Affiliations:** School of Chemical, Petroleum and Gas Engineering, Iran University of Science and Technology, Narmak, Tehran, 16846 Iran

**Keywords:** Hydrogen production, Dimethyl ether, Steam reforming, Partial oxidation, Response surface methodology (RSM), Artificial neural network (ANN), Chemical engineering, Energy science and technology

## Abstract

This study aims to develop two models for thermodynamic data on hydrogen generation from the combined processes of dimethyl ether steam reforming and partial oxidation, applying artificial neural networks (ANN) and response surface methodology (RSM). Three factors are recognized as important determinants for the hydrogen and carbon monoxide mole fractions. The RSM used the quadratic model to formulate two correlations for the outcomes. The ANN modeling used two algorithms, namely multilayer perceptron (MLP) and radial basis function (RBF). The optimum configuration for the MLP, employing the Levenberg–Marquardt (trainlm) algorithm, consisted of three hidden layers with 15, 10, and 5 neurons, respectively. The ideal RBF configuration contained a total of 80 neurons. The optimum configuration of ANN achieved the best mean squared error (MSE) performance of 3.95e−05 for the hydrogen mole fraction and 4.88e−05 for the carbon monoxide mole fraction after nine epochs. Each of the ANN and RSM models produced accurate predictions of the actual data. The prediction performance of the ANN model was 0.9994, which is higher than the RSM model's 0.9771. The optimal condition was obtained at O/C of 0.4, S/C of 2.5, and temperature of 250 °C to achieve the highest H_2_ production with the lowest CO emission.

## Introduction

Global population expansion and urbanization have led to enormous increases in energy demand, primarily reliant on fossil fuels. This has resulted in higher quantities of CO_2_ and other greenhouse gases (GHGs), causing global warming. Therefore, decarbonizing the energy supply with clean, sustainable, and renewable energy is vital for long-term viability and global safety^1,2^. Hydrogen has garnered significant interest among several alternative fuels as a prospective clean energy source. Hydrogen, unlike fossil fuels, is not easy to find in nature. On the other hand, it can be made from any main energy source and used as fuel in either an internal combustion engine or a fuel cell. It just generates water as a byproduct. Hydrogen is the only known fuel that doesn't contain carbon and has the highest energy density of any known fuel. Because of this, it is widely seen as an environmentally friendly alternative to fossil fuels. Another benefit is that hydrogen can be used at home with the right storage technologies. This is because normal methods can safely transport it, and it can be stored as either a compressed gas, a cryogenic liquid, or a solid hydride^3,4^. The investigation of discernible sustainable hydrogen sources from various feedstocks, namely methanol, ethanol, and dimethyl ether (DME), presented in Table 1, unveiled a notable limitation: the temperatures essential for hydrogen production from methanol and ethanol were considerably elevated compared to those necessary for employing DME. In addition, dimethyl ether (DME) is a promising fuel for generating hydrogen-rich fuel cells due to its high hydrogen-to-carbon ratio, high energy density, and non-toxic properties. As with liquefied petroleum gas (LPG), it can be easily handled, stored, and transported, and LPG's equipment can be easily adapted for DME due to its similar physical properties^5^. DME has lower NO_x_ and SO_x_ pollutants than traditional diesel and is non-carcinogenic, non-teratogenic, and non-mutagenic. It burns with a visible blue flame and produces the least greenhouse gas (GHG) emissions compared to other fuels. It has the highest well-to-wheel efficiencies of all non-petroleum-based fuels using conventional, hybrid, and fuel processor fuel cell vehicle technologies^6^. Furthermore, regarding economic viability and thermodynamic preference, the direct conversion of syngas into DME is superior to the synthesis of methanol^7^. There are three primary processes for the production of hydrogen-rich fuel cell feeds using DME: steam reforming (SR), partial oxidation (PO), and auto-thermal reforming (ATR)^8^. Most research papers primarily concentrate on SR, whereas there are a limited number of articles that specifically address PO and ATR^9^. Takeishi et al. investigated various copper-alumina catalysts for generating hydrogen from dimethyl ether (DME). Among these catalysts, the Cu–Zn (29–1 wt%)/Al_2_O_3_ catalyst exhibited the highest hydrogen production and the lowest CO production from DME. When subjected to DME steam reforming at 275 °C, the catalyst achieved a DME conversion rate of 95%, an H_2_ yield of 95%, and a CO concentration of 0.8% mol^10^. However, the operational cost may increase because the SR is an endothermic process and requires a significant amount of energy. Catalytic PO is an exothermic reaction that can be coupled with an endothermic reaction to produce a valuable product. The catalytic PO's exothermic properties enable the utilization of a more compact reactor design and accelerate the initiation of reactions, presenting an encouraging advancement in technology^9,11^.

**Table 1:** Several studies have been done on the optimization of hydrogen production from steam reforming.

Researcher	Catalyst	BET (m^2^/g)	T(°C)	Conversion			H_2_ Yield	References
				DME	Ethanol	Methanol		
Takeishi et al	ƔAl_2_O_3_(Sol)//CuZn(9 wt.%)/SiO_2_(Sol) (Two-layer type)	368	300	58.6	–	–	27.2	^10^
Takeishi et al	ƔAl_2_O_3_(Sol)&CuZn(9–1wt.%)/SiO_2_(Sol) (Mixed type)	368	300	88.4	–	–	76.6	^10^
Takeishi et al	Cu–Zn(4.5–0.5 wt.%)/Al_2_O_3_(Sol) (Single-use type)	237	300	91.9	–	–	88.8	^10^
Wang et al	15 wt.% Ni/zeolite	–	550	–	100	–	76.0	^13^
Musso et al	15 wt.% Ni/Y-ZrO_2_	–	650	–	100	–	91.0	^14^
Prasongthum et al	10 wt.% Ni/CNTs–SF	–	–	–	100	–	40.0	^15^
Lorenzut et al	Ni/Cu/ZnO/Al_2_O_3_	–	350	–	–	100	83.0	^16^
Qi et al	NiAl-LDH (Ni/Al = 4.9)	–	390	–	–	94.6	70.0	^17^

**Figure 1:**
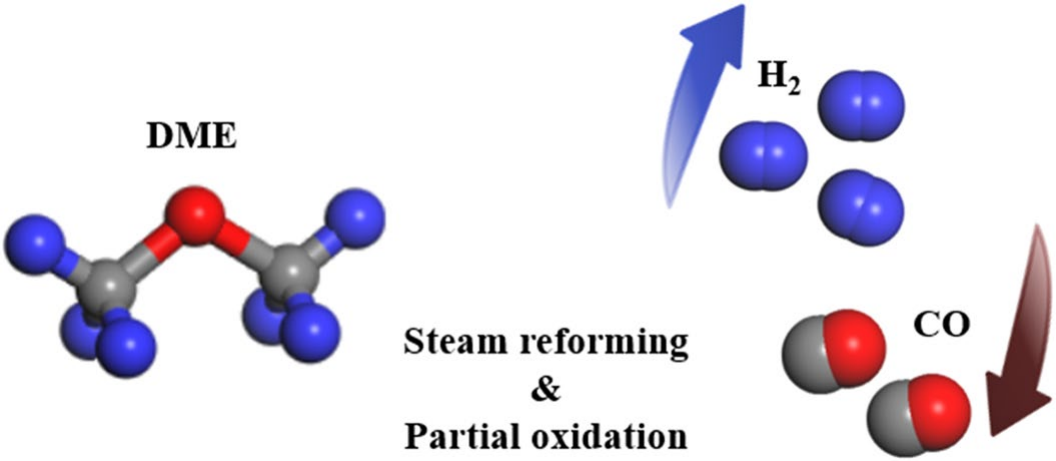
H_2_ production from DME SR and PO.

For specific requirements, the combined process can be optimized through the modification of operational conditions. Combining the SR and PO processes to generate hydrogen-rich fuel cell feeds with DME gives more control over the hydrogen-to-CO (H_2_/CO) ratio, which leads to a higher hydrogen yield compared to using just one process (Fig. 1). The increased fraction of fully oxidized DME also helps lower the amount of carbon monoxide (CO), which improves the performance of fuel cells^11,12^.

Operating auto thermally, the energy sufficient to propel the SR reaction is provided by the PO reaction. The highest efficiency for hydrogen production is achieved through SR, whereas complete oxidation results in zero efficiency. As the process shifts from pure SR to oxidation, the production efficiency of hydrogen and the amount of CO decrease. The global minimum of CO is achieved with full oxidation, although this approach is not feasible when the intended outcome is hydrogen. If only heat energy is required, complete oxidation can be employed for rapid heating. However, for the purpose of hydrogen production, the local minimum of CO ensues under the particular conditions of pure steam reforming. Optimal processing conditions for hydrogen production efficiency, CO reduction, and total energy efficiency occur in the zone between PO and SR under steady-state conditions and without sudden heating^18^.

The integration of RSM and ANN in the optimization of hydrogen production through DME steam reforming and partial oxidation indeed holds significant promise for enhancing efficiency and sustainability in various industries. By leveraging these advanced modeling and optimization techniques, researchers can effectively fine-tune the process parameters involved in DME steam reforming and partial oxidation to achieve optimal hydrogen yields while simultaneously minimizing energy consumption and environmental impact. RSM provides a systematic and efficient approach for exploring the relationship between input variables (such as temperature, pressure, and catalyst composition) and the output response (hydrogen yield). Through the design and analysis of experiments, RSM enables the generation of response surfaces that depict the relationship between the input variables and the desired output, guiding the search for optimal operating conditions. The combined use of RSM and ANN allows for a comprehensive optimization strategy, wherein Design Expert guides the experimental design and data collection process, while ANN aids in model development and prediction. Pardo et al. investigated methods to enhance the operational performance of a real steam reforming plant, with a specific focus on increasing hydrogen production or maximizing factory profits. In their study, they constructed a holistic model of the SR plant comprising seven multilayer perceptrons (MLPs) and utilized genetic algorithm (GA) and memetic algorithm (MA) optimization techniques to refine model input^19^. Zhang et al. have designed an optimal model predictive control strategy for hydrogen production. This study involves a thermodynamic assessment of hydrogen production through low-temperature auto-thermal reforming (ATR) of DME. The experimental and simulated demonstrations of the Pd/Zn/γ-Al_2_O_3_ catalyst, which is applied to honeycomb cordierite ceramic, revealed its effectiveness at 400 °C^20^. Chen et al. conducted a study where they utilized ANN to forecast methanol conversion and H_2_ yield by incorporating input variables such as the steam-to-carbon (S/C) ratio, gas hourly space velocity (GHSV), and reaction temperature^21^. Lghalo et al. explored the thermodynamic analysis of dimethyl ether (DME) steam reforming in conjunction with a statistical approach. Employing Response Surface Methodology (RSM), they investigated potential interactions among various process factors. They developed regression models to forecast the percentage molar yield of each species, considering key factor ranges such as temperature (450–750 °C), pressure (1–5 atm), and steam-to-carbon (S/C) ratio (1–5)^22^.

Previous studies (Table 2) in the literature have separately investigated the SR and PO processes, but the application of RSM and ANN to optimize hydrogen production via DME steam reforming and partial oxidation has not been explored. This research aims to utilize RSM and ANN, such as the multi-layer perceptron (MLP) and radial basis function (RBF) network approaches, to model thermodynamic data on hydrogen production from the combined processes of DME SR and PO.

**Table 2:** Utilizing simulations and optimization techniques to enhance hydrogen production via DME steam reforming.

Researcher	GHSV-1 (h^− 1^)	T (°C)	P (bar)	O/C	S/C	H_2_ yield *R*^2^ (RSM)	H_2_ yield *R*^2^ (ANN)	References
Pardo et al	–	423.9–455	–	–	2.74–4	–	0.9848	^19^
Chen et al	15,000–30,000	180–280	–	–	1.5–2–2.5	0.9842	0.99	^21^
Ighalo et al	–	450–750	1–5	–	1–5	0.9914	–	^22^

However, this research has specifically concentrated on optimizing the integration of these two processes to achieve maximum H_2_ production and minimize CO production. The independent variables considered include the oxygen-to-carbon ratio (O/C), steam-to-carbon ratio (S/C), and temperature (T), with the hydrogen mole fraction (${y}_{{\text{H}}_{2}}$) and CO mole fraction (${y}_{\text{CO}}$) as the response of the modeling approach. The impact of operating pressure on hydrogen content is found to be minimal, so all computations are conducted under atmospheric pressure conditions (*P* = 1 atm). The RSM modeling method aims to find the best conditions and create a semi-empirical model that fits the data best by looking at how different variables affect ${y}_{{\text{H}}_{2}}$ and ${y}_{\text{CO}}$. In the same way, the ANN approach aims to determine the best network configuration and the most appropriate weights and biases for examining the correlation between ${y}_{{\text{H}}_{2}}$ and ${y}_{\text{CO}}$ and the independent variables by determining the network's specifications, such as hidden layer size, number of neurons, and training functions for the MLP network and the number of neurons, spread value, and epoch for the RBF network. The study also shows how design and statistical models can make the process more efficient. The RSM method uses thermodynamic data to fit a quadratic model, and the ANN method develops models based on thermodynamic data and does sensitivity analysis to see which input parameters are most important. The correlations facilitate the timely estimation of process yield, thereby influencing the selection of various design alternatives. Moreover, they bear significance in budgeting, forecasting, and other facets of process design and economics.

## Methodology

### Thermodynamic equilibrium calculations

The selected reactions for each of the exothermic and endothermic steps based on the combined mechanism of DME SR and PO for the primary thermodynamic equilibrium calculations are listed in Table 3:

**Table 3:** Selected reactions for each step of the process.

Reaction	Equation	∆H°_rxn_ (kJmol − 1)
DME PO	CH_3_OCH_3_ + 0.5O_2_ $\rightleftharpoons$ 2CO + 3H_2_	− 25
DME oxidation:	CH_3_OCH_3_ + 3O_2_ $\rightleftharpoons$ 2CO_2_ + 3H_2_O(v)	− 1316
DME SR	CH_3_OCH_3_ + 3H_2_O(v) ⇌ 2CO_2_ + 6H_2_	+ 135
Water–gas shift	H_2_ + CO_2_ ⇌ H_2_O(v) + CO	+ 41

After selecting reactants, products, and operating conditions (i.e., the O/C ratio, S/C ratio, and temperature), equilibrium compositions were calculated through the minimization of Gibb's free energy, using the Peng-Robinson equation as the equation of state and Aspen Tech software for multicomponent equilibria. The equilibrium compositions were subsequently charted for various processing conditions to ascertain the most favorable processing temperature and feed composition^18^.

### Data collection

The data required to train and test the models was gathered from previous studies in the literature^12^, as this study is exclusively computational. A total of 624 data points were collected from the literature (supplementary Table S2). Out of them, 591 were utilized for training and 33 for testing. Table 4 presents some of the thermodynamic data points that pertain to the mole fraction of hydrogen and CO under different operating conditions.

**Table 4:** Some thermodynamic data are used for RSM and ANN modeling ^17^.

Operating conditions			Mole fraction	
O/C	S/C	T (°C)	H_2_	CO
0.40	2.50	300	0.4375	0.0071
2.00	0.75	500	0.1265	0.0160
0.00	1.25	200	0.7246	0.0693
0.80	1.50	500	0.3516	0.0475
1.20	2.50	400	0.2388	0.0081
1.60	1.50	500	0.1816	0.0179
1.60	3.00	600	0.1494	0.0151
0.40	1.50	300	0.5154	0.0223
2.40	2.25	400	0.0646	0.0018
0.80	0.50	300	0.4100	0.0679

### RSM theory

Experimental design is a scientific methodology that entails manipulating input variables to acquire knowledge about the process and establish a desired input–output correlation. This approach has multiple advantages, including the ability to identify the pivotal aspects that impact the process, decrease the process's expenses, construct a model of the process, and ascertain the correlation between input and output variables^23^. RSM is a statistical modeling technique that uses quantitative data from well-designed experiments to answer numerous regression equations simultaneously^24^. It models the correlation between independent and dependent variables as a surface^25^. The model aims to establish a relationship, even approximate, between Y and control variables, determine the significance of factors represented by *x*_1_, *x*_2_,…, *x*_k_ through hypothesis testing, and determine the optimal settings for maximum or minimum response in a specific region^26^. Equation 1 represents the quadratic model in the RSM method.

1 $$ Y = \beta_{0} + \mathop \sum \limits_{j = 1}^{k} \beta_{j} x_{j} + \mathop \sum \limits_{j = 1}^{k} \beta_{jj} x_{j}^{2} + \mathop \sum \limits_{i} \mathop \sum \limits_{j > = 2}^{k} \beta_{ij} x_{i} x_{j} + e_{i} $$

where the predicted response is denoted as *Y* (i.e., the mole fraction of hydrogen or CO); *x*_i_ and *x*_j_ are independent variables (*i* and *j* vary from 1 to k); *β*_0_ is the model intercept coefficient; The interaction coefficients for the linear, quadratic, and second-order terms are represented as *β*_j_, *β*_jj_, and *β*_ij_, respectively; *k* is the number of independent parameters; whereas *e*_i_ is the error^27^. In the RSM method, the experimental data is used to fit the quadratic model described in Eq. (1), and the coefficients of the model are determined. The accuracy of the resulting model is assessed through an analysis of variance (ANOVA), the correlation coefficient (*R*^2^), and the model's *p*-value. Table 5 summarizes the values of the independent parameters, organized according to their symbol assignment, response, and trial range.

**Table 5:** The hydrogen production process parameters and responses.

Parameters	Type	Units	Symbol	Ranges
Oxygen-to-carbon ratio (O/C)	Input	–	A	0–2.8.0
Steam-to-carbon ratio (S/C)	Input	–	B	0.25–4.0
Temperature (T)	Input	℃	C	100–600
Hydrogen mole fraction (${\text{y}}_{{{\text{H}}_{2} }}$)	Response	–	${\text{y}}_{{{\text{H}}_{2} }}$	0.007–0.7246
Carbon monoxide mole fraction (${\text{y}}_{{{\text{CO}}}}$)	Response	–	${\text{y}}_{{{\text{CO}}}}$	0–0.3333

### ANN theory

The ANN method has become more popular over the last two decades because it seeks to model computer networks like the way the human brain and nerves are structured. Mathematicians and computer scientists created ANNs to imitate real-world neural networks. ANNs are mathematical tools or computational models that process information using a connectionist approach. They offer advantages such as high processing speed, input–output data connection, network compatibility, noisy data response, parallel processing ability, fault tolerance, and learning. ANN is typically an adaptive system that can learn from available data and map input parameters to output parameters without knowing the complex relationship between them. This adaptability is accomplished by changing the structure of the system in response to external or internal information acquired during the learning phase. Modern statistical data models use non-linear neural networks. They are frequently used to simulate complicated input–output interactions or uncover data patterns^28,29^. ANNs, consisting of three layers: input, hidden, and output, are created by interconnected neurons. The structure of these networks is determined by weight parameters and activation functions. Throughout the learning process, a dedicated training algorithm optimizes weights and bias values, aiming to minimize error values between real parameter values and the projected values by the ANN^23,30^. Neurons are the tiniest units that process data. A cell gathers information from several sources. The inputs (*x*_i_) are multiplied by their corresponding weights (*w*_i_), the resulting values are summed, and the bias vector (*b*) is added to the overall sum. This process for input data is summarized in Eq. (2).

2 $$ net = \left( {\mathop \sum \limits_{i = 1}^{n} w_{i} x_{i} } \right) + b $$

This process ends with the results fed into a transfer function (*f*), and the output values (*y*) are derived from the following equation^31^.

3 $$ y = f\left( {net} \right) $$

In order to get acceptable outcomes from the neural network, it is important to normalize the data. To achieve this objective, all data have been normalized within the range of 0 to + 1 using Eq. (4):

4 $$ {\text{ X}}_{{{\text{norm}}}} = \frac{{2X - X_{Max} - X_{Min} }}{{X_{Max} - X_{Min} }} $$

where X_norm_ represents the normalized data, X is the input variable, and *X*_max_ and *X*_min_ correspond to the maximum and minimum values of the data, respectively^32^. To determine network parameter values, the projected network error should be kept to a minimum for every step of the mean square error (MSE) in every iteration during network training. MSE, mean absolute error (MAE), mean absolute percentage error (MAPE), the square of the correlation coefficient (*R*^2^), and the average absolute relative deviation (AARD%) are used to compare the model results to the validation dataset. *R*^2^ close to 1 and near-zero MSE, MAE, and MAPE values imply network accuracy. MSE, MAE, MAPE, *R*^2^, and AARD are obtained as follows^32–34^:

5 $$ MSE = \frac{1}{n}\mathop \sum \limits_{i = 1}^{n} \left( {Y_{predicted} - Y_{actual} } \right)^{2} $$

6 $$ MAE = \frac{1}{n}\mathop \sum \limits_{i = 1}^{n} \left| {Y_{actual} - Y_{predicted} } \right| $$

7 $$ MAPE = \frac{100}{n}\mathop \sum \limits_{i = 1}^{n} \frac{{\left| {Y_{actual} - Y_{predicted} } \right|}}{{Y_{actual} }} $$

8 $$ R^{2} = \frac{{\mathop \sum \nolimits_{i = 1}^{n} \left( {Y_{predicted} - Y_{actual} } \right)^{2} }}{{\mathop \sum \nolimits_{i = 1}^{n} \left( {Y_{predicted} - Y_{mean} } \right)^{2} }} $$

9 $$ {\text{AARD}} = \frac{1}{n}\mathop \sum \limits_{i = 1}^{n} \left| {\frac{{Y_{actual} - Y_{predicted} }}{{Y_{actual} }}} \right| \times 100 $$

where *Y*_*predicted*_, *Y*_*actual*_, *Y*_*mean*_, and n denote the predicted *Y* value using a neural network, the actual Y value, the average Y value, and the number of data points, respectively.

#### MLP model

MLP is a type of neural network composed of numerous layers of simple, two-state, sigmoid processing components (nodes) or neurons that interact with one another through weighted connections. Following a lowermost input layer, there are normally several intermediate or hidden layers, followed by a top output layer. Nevertheless, every neuron within a layer is intricately linked to the neurons in adjacent layers. There are no connections between elements in a single layer. Weights determine the extent of interaction among the activity levels of interconnected neurons^35^. MLP uses the backpropagation method as a feed-forward neural network, whereby the errors are propagated backward to modify the weights and arrange data into appropriate outputs. It is a more advanced variant of the standard linear perceptron that can gather information from non-linearly separable input^36^. Based on the chosen training algorithm, weights and bias values are adjusted in each epoch, attaining optimal weight and bias values. The selection of the number of hidden layers and neurons in the MLP network is done through trial and error. By evaluating the MSE values, it is possible to determine the network's optimal structure by adjusting the number of neurons and the size of the hidden layers^37^. The computational basis of the MLP network is expressed in Eq. (10).

10 $$ y = f_{2} \left( {\mathop \sum \limits_{j = 1}^{n} w_{j} f_{1} \left( {\mathop \sum \limits_{i = 1}^{n} h_{ij} x_{i} + b_{j} } \right) + b_{0} } \right) $$

**Figure 2:**
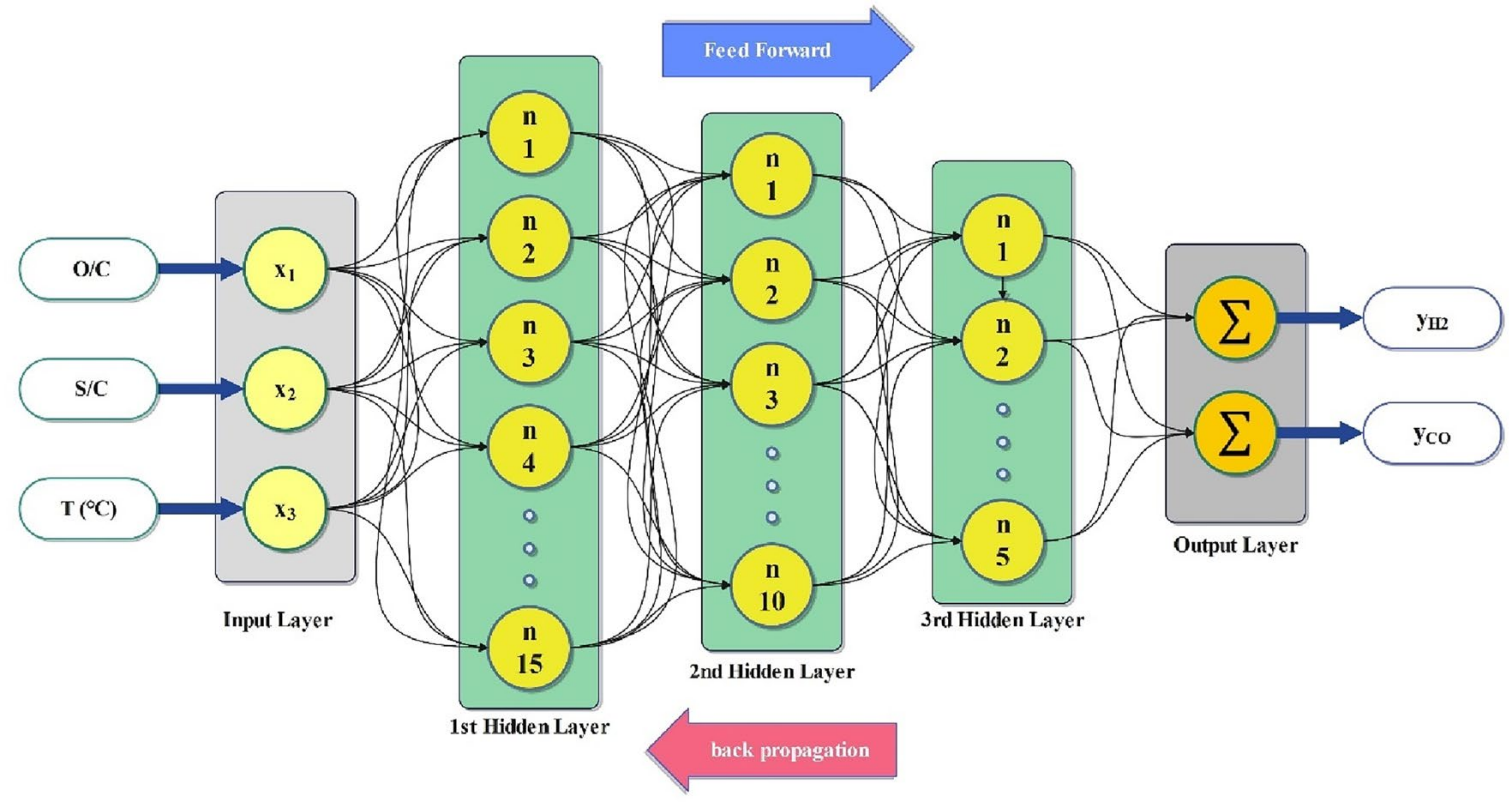
Schematic diagram of the MLP network with three hidden layers.

The weight matrix, bias vector, and activation function of the hidden layer are denoted as *h*_ij_, *b*_j_, and *f*_1_, respectively. Similarly, the weight, bias vector, and activation function of the output layer are represented as *w*_j_, *b*_0_, and f_2_^38^. The structure of an MLP with three hidden layers is depicted in Fig. 2. During the training phase of the network, the objective is to minimize MSE by producing an error signal derived from the activation function. Subsequently, the weights are then adjusted by propagating this error signal backward through the layers. Weights that lower MSE are preferable.

#### RBF model

**Figure 3:**
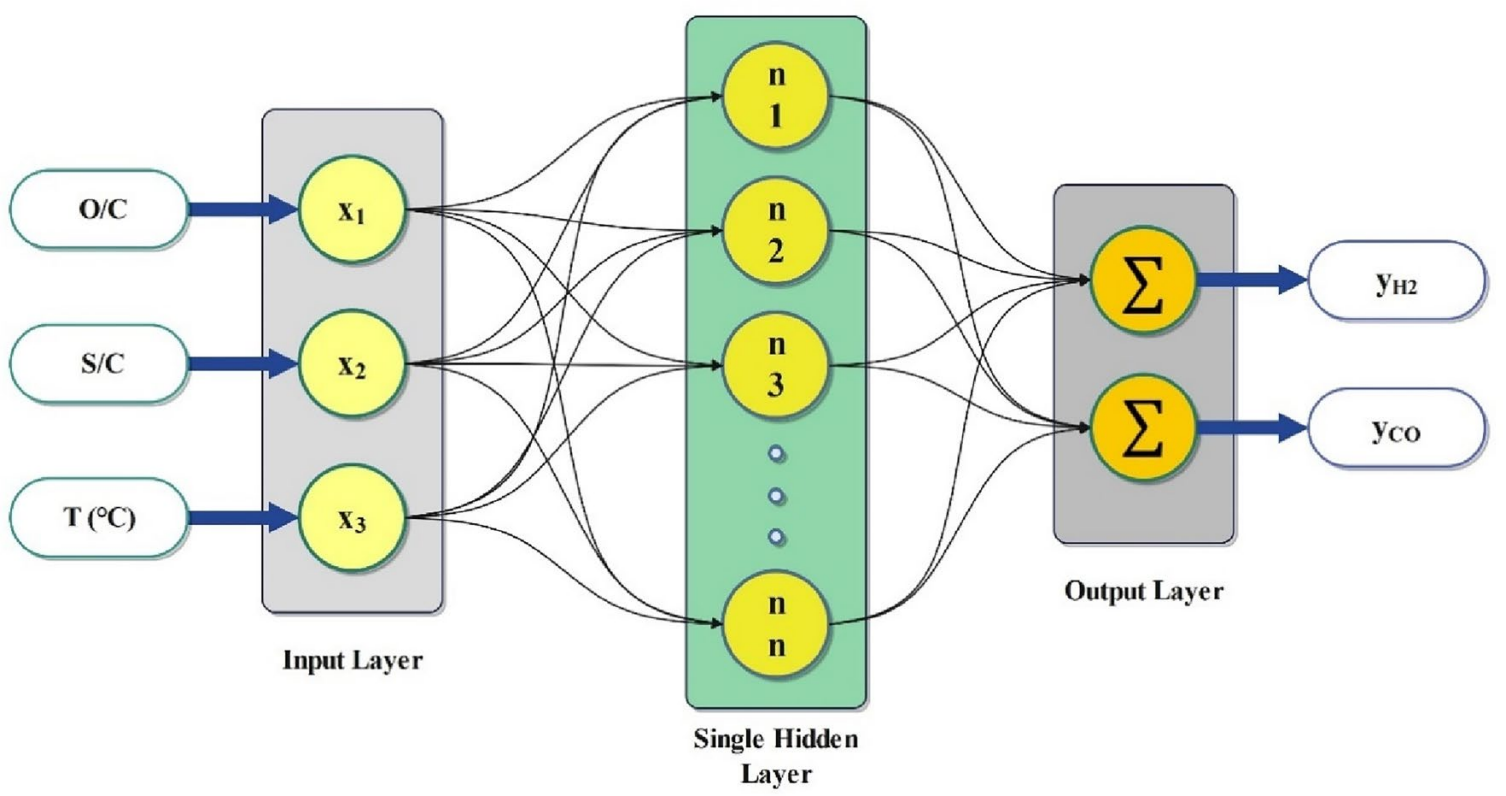
Schematic diagram of the RBF network with a single hidden layer.

RBF networks have been demonstrated to be general approximates of a subclass of single-hidden-layer feedforward neural networks^39^. This network, developed by Broomhead and Lowe, is widely used in complex dynamic system modeling based on its exceptional capabilities for nonlinear mapping, self-organization, generalization, and self-learning^40^. The RBF network is composed of three layers: the input layer, the hidden layer, and the output layer. The input layer is tasked with processing a substantial quantity of non-linear data, which is then sent to the hidden layer^41^. The hidden layer contains a variety of neurons, and the optimal number of neurons is determined by trial and error. The basic concept of the learning process of these networks is to minimize the MSE until it reaches a certain value (e.g., 0.0001) or a specified number of epochs (e.g., 100 epochs)^42^. In the RBF network, the Gaussian function, defined by Eq. (11), is the most commonly utilized activation function for processing data.

11 $$ {\Phi }_{i} \left( {x - C_{i} \times b} \right) = exp\left( { - \frac{{\left( {x - C_{i} \times b} \right)^{2} }}{{2{\upsigma }_{i}^{2} }}} \right) $$

where *x* is the input variable, *c*_*i*_ is the center point, *b* is the bias, and *σ*_*i*_ is the spread of the Gaussian function^43^, and Eq. (12) shows the general form of the computation basis in the RBF network.

12 $$ y = \mathop \sum \limits_{i = 1}^{n} w_{i} {\Phi }_{i} $$

where the output of the network is denoted by *y*, the Gaussian function is represented by Φ_*i*_, the number of hidden layer neurons is indicated by n, the linkage weight between the *i* neuron and the output layer is represented by *w*_i_, and the || shows the Euclidean norm^44^. Figure 3 shows the structure of the RBF network with a single hidden layer used in this work.

#### ANN structure design

**Figure 4:**
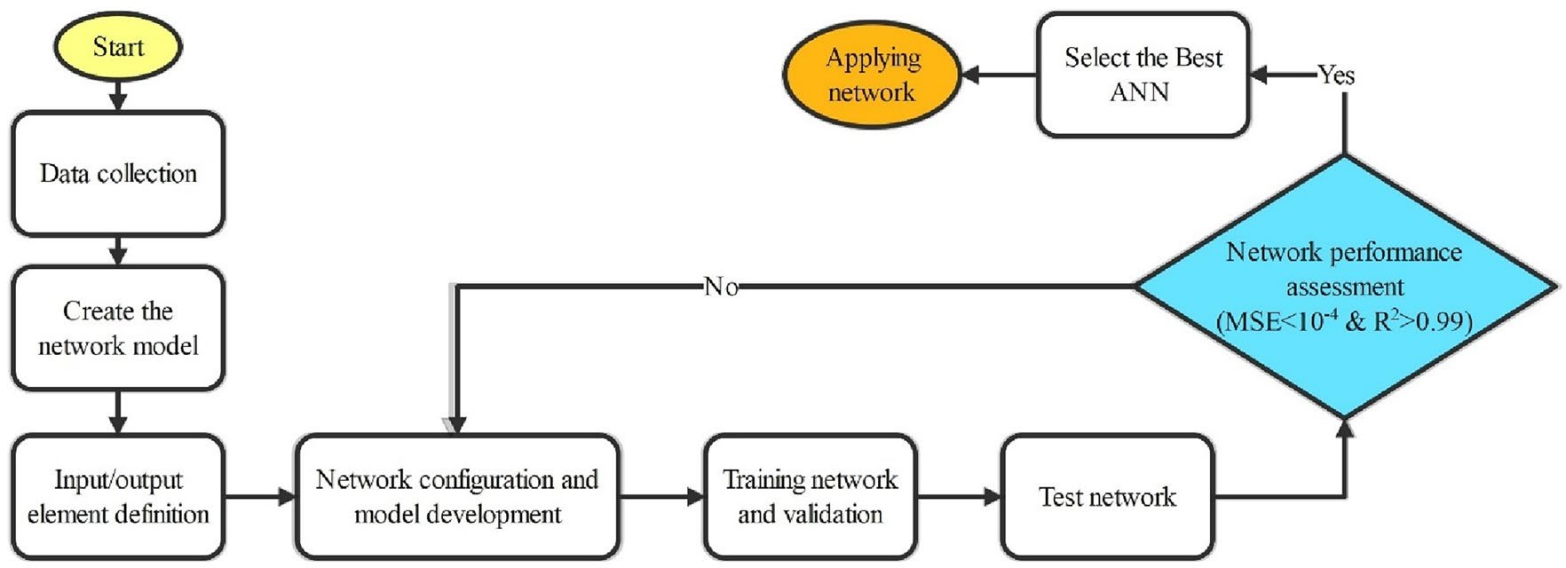
Schematic flowchart of ANN structure design.

A flowchart illustrating the ANN model design procedure is shown in Fig. 4. In the first step, we gather data from the literature. The thermodynamic and process parameters such as S/C, O/C, and temperature were used as input data, and ${y}_{{H}_{2}}$ or ${\text{y}}_{CO}$ were employed as output data. In the next step, the model is created by defining the input and output elements. Following that, the input and output data were normalized before selecting the learning algorithm for developing the network structure. In the subsequent stage, the ANN model training approach and network training validation are used to match the data and set the training, which includes adjusting the inputs and outputs of the network. The chosen model is trained using a certain data set to maximize its performance by adjusting network parameters such as weight, bias, or threshold. The network validation data is supplemented by including a specific dataset during the training phase, whereas the network testing is performed using the selected test data. Once generalizations have been optimized, the training process is complete. To evaluate the accuracy of the trained model, statistical metrics such as *R*^2^ value and MSE were used to compare the model's predictions with the actual data. The MLP model's optimum configuration was discovered by variation with different numbers of hidden layers, neurons within each layer, and network training algorithms to get the most accurate predictions. In contrast, the RBF network typically determines the number of neurons by an iterative process of trial and error. Initially, many neurons are used in the single hidden layer, and then the number is gradually reduced until the least MSE is achieved. The decrease in the number of neurons leads to a reduction in the proportional error. The algorithm's training procedure ends upon achieving the optimized error from the testing data.

## Result

### RSM results

This section analyzes several fitting models and their ability to match thermodynamic data. Based on its optimum *R*^2^ value, the quadratic model without a transfer function was selected as the most accurate model. In addition, the dependence of hydrogen production on variable parameters and RSM-based optimization of hydrogen production were discussed.

#### Analysis of variance (ANOVA)

The ANOVA results from the thermodynamic data analysis are shown in Table 6. The F-value determines the model's overall significance. The *p*-value shows the probability involved with the ANOVA analysis. The parameters of the model are considered statistically significant if their *p*-values are lower than 0.05^45^. Conversely, *p*-values higher than 0.1 indicate that the parameters are not statistically significant^46^. Based on the results of this research, the ${\text{y}}_{{\text{H}}_{2}}$ and ${\text{y}}_{\text{CO}}$ models had significant F-values of 7713.94 and 1038.29, respectively. The *p*-values for the model term (< 0.0001) indicate that the models are significant and reliable. Between the independent variables, parameters A, B, C, AB, A^2^, and C^2^ for ${\text{y}}_{{\text{H}}_{2}}$ and all parameters for ${\text{y}}_{\text{CO}}$ are significant. The term A has the most impact on the ${y}_{{\text{H}}_{2}}$ because it has the greatest value of F and the lowest value of *p* > F. Moreover, term C, which has the greatest value of F and the lowest value of *p* > F, has the most impact on ${y}_{\text{CO}}$. The Adeq precision signal-to-noise ratio values of the ${\text{y}}_{{\text{H}}_{2}}$ and ${\text{y}}_{\text{CO}}$ models are 353.2301 and 162.0399, respectively, significantly above 4. This indicates that the model developed can be used for industrial process design. Fit statistic parameters such as *R*^2^, predicted *R*^2^, and adjusted *R*^2^ for the responses are listed in Table 7.

**Table 6:** ANOVA results for two studied responses, ${\text{y}}_{{{\text{H}}_{2} }}$ and ${\text{y}}_{{{\text{CO}}}}$.

	$y_{{H_{2} }}$					$y_{CO}$				
	Sum of Squares	df	Mean Square	F-value	*p*-value	Sum of Squares	df	Mean Square	F-value	*p*-value
Model	6.630	9	0.7363	7713.94	< 0.0001	0.0371	9	0.0041	1038.29	< 0.0001
A-O/C	4.290	1	4.290	44,899.4	< 0.0001	0.0108	1	0.0108	2727.15	< 0.0001
B-S/C	0.1569	1	0.1569	1643.64	< 0.0001	0.0044	1	0.0044	1119.37	< 0.0001
C-T	0.0103	1	0.0103	108.07	< 0.0001	0.0198	1	0.0198	4976.15	< 0.0001
AB	0.0653	1	0.0653	684.29	< 0.0001	0.0021	1	0.0021	534.85	< 0.0001
AC	7.6e−6	1	7.6e−6	0.0801	0.7774	0.0098	1	0.0098	2480.01	< 0.0001
BC	0.0002	1	0.0002	2.020	0.1557	0.0015	1	0.0015	372.66	< 0.0001
A^2^	0.2007	1	0.2007	2103.18	< 0.0001	0.0008	1	0.0008	203.36	< 0.0001
B^2^	1.2e−7	1	1.2e−7	0.0012	0.9719	0.0002	1	0.0002	55.73	< 0.0001
C^2^	0.0353	1	0.0353	370.27	< 0.0001	0.0006	1	0.0006	162.93	< 0.0001
Residual	0.0372	390	0.0001	–	–	0.0015	390	3.9e−6	–	–
Cor Total	6.660	399	–	–	–	0.0386	399	–	–	–

**Table 7:** The statistical parameters of the quadratic model are employed to model responses.

Statistic parameters	$y_{{H_{2} }}$	$y_{CO}$
R^2^	0.9944	0.9599
Adjusted R^2^	0.9943	0.9590
Predicted R^2^	0.9940	0.9571
Adeq Precision	353.2301	162.0399

#### RSM Correlations

The thermodynamic data were modeled using the quadratic model equation presented in Eqs. (13) and (14). The model showed the impact and interactions of O/C, S/C, and temperature on ${y}_{{\text{H}}_{2}}$ and ${y}_{\text{CO}}$.

13 $$ \begin{aligned} y_{{H_{2} }} & = \, 0.{6}0{5341 } + \, - 0.{346977 } \times {\text{ A }} + \, - 0.0{56851 } \times {\text{ B }} + \, 0.000{351321 } \times {\text{ C }} + \, 0.0{2}0{281 } \times {\text{ AB }} \\ &\quad+ { 1}.{39}0{\text{33e}} - 0{6 } \times {\text{ AC }} + { 4}.{\text{64555e}} - 0{6 } \times {\text{ BC }} + \, 0.0{415316 } \times {\text{ A}}^{{2}}  + { 1}.{\text{59334e}} - 0{5 } \times {\text{ B}}^{{2}} \\ &\quad + \, - {4}.{52}0{\text{12e}} - 0{7 } \times {\text{ C}}^{{2}} \end{aligned} $$

14 $$ \begin{aligned} y_{CO} & = \, - 0.000{465333 } + \, - 0.00{7}0{8278 } \times {\text{ A }} + \, - 0.00{8}0{5}00{3 } \times {\text{ B }} + \, 0.000{119}0{38 } \times {\text{ C }} \\ &\quad + \, 0.00{365635 } \times {\text{ AB }} + \, - {4}.{\text{98974e}} - 0{5 } \times {\text{ AC }} + \, - {1}.{\text{28554e}} - 0{5 } \times {\text{ BC }} \\ &\quad + \, 0.00{263349 } \times {\text{ A}}^{{2}} + \, 0.000{688595 } \times {\text{ B}}^{{2}} + { 6}.{\text{11429e}} - 0{8 } \times {\text{ C}}^{{2}} \end{aligned} $$

A positive value in coded equations signifies the factor that leads to optimization, while a negative value indicates an inverse link between responses and variables. The software prefers the quadratic model for the responses.

#### Diagnostics graphs

**Figure 5:**
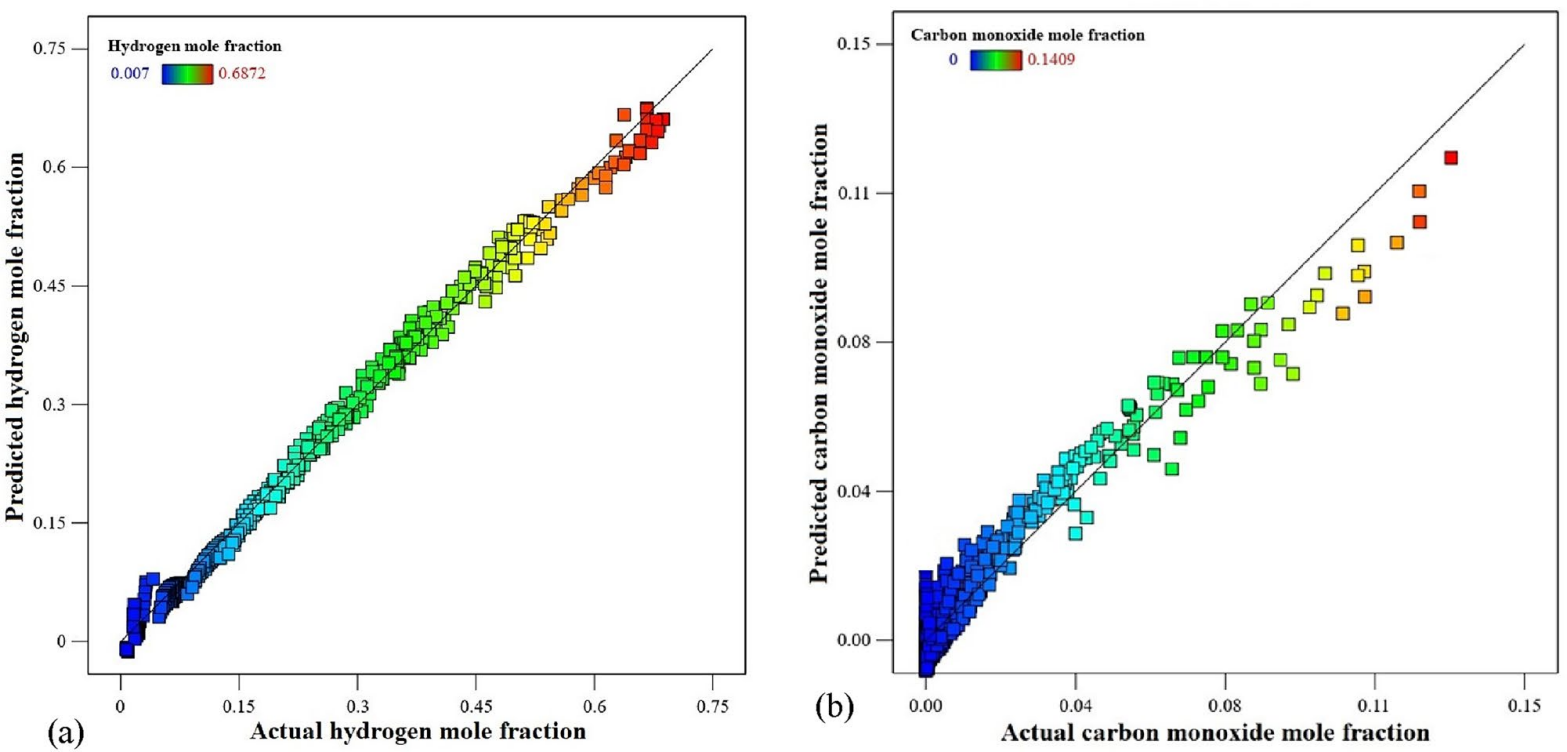
Predicted versus actual graphs for two responses of (**a**) $y_{{H_{2} }}$ and (**b**) $y_{CO}$.

Diagnostic graphs are another way to determine a model's validity. As shown in Fig. 5(a,b), the quadratic models that were employed to predict the responses of ${y}_{{\text{H}}_{2}}$ and ${y}_{\text{CO}}$ were assessed by contrasting the predicted and actual data. The linear data distribution in Fig. 5 and the statistical parameters in Table 7 demonstrated that the quadratic models matched the thermodynamic data for both responses quite well.

#### Perturbation graphs

**Figure 6:**
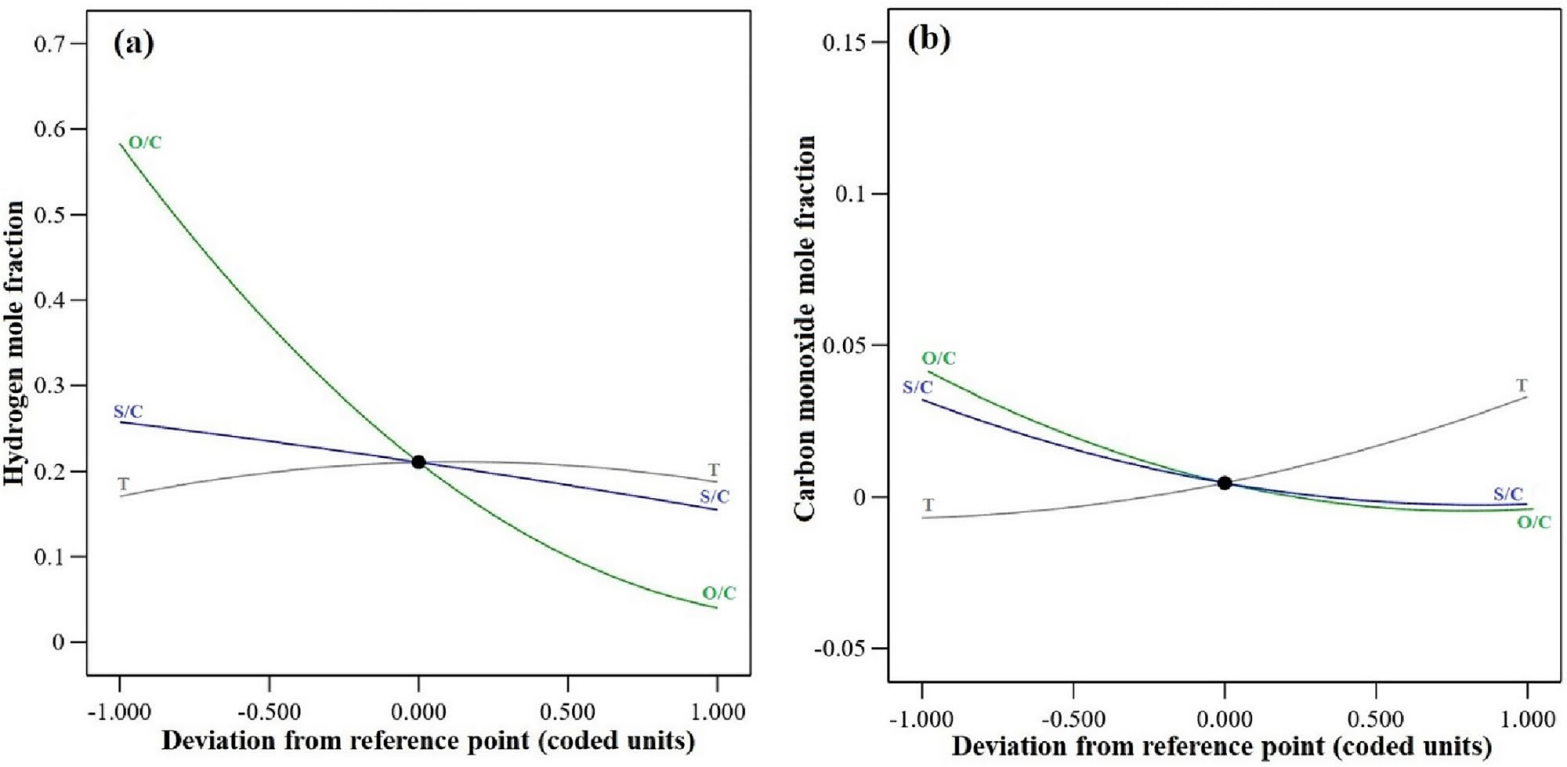
Perturbation graphs for (**a**) $y_{{H_{2} }}$, (**b**) $y_{CO}$.

The perturbation graphs depict the impact of all parameters on response performance, with the operating midpoint range as the center point. The graphs compare the impact of all factors on ${\text{y}}_{{\text{H}}_{2}}$ and ${\text{y}}_{\text{CO}}$ at the center point, allowing for easy observation of each factor's impact. The perturbation scheme for comparing all operating parameters is shown in Fig. 6(a,b). ${y}_{{\text{H}}_{2}}$ shows a significant drop when O/C increases, whereas S/C and temperature have a less pronounced effect. As the S/C ratio increases, ${y}_{{\text{H}}_{2}}$ decreases gradually; while an increase in temperature initially results in a slight rise in ${y}_{{\text{H}}_{2}}$, it subsequently leads to a consistent reduction in ${y}_{{\text{H}}_{2}}$. Conversely, ${y}_{\text{CO}}$ drops gradually as O/C and S/C increase, whereas rising temperatures show a steady rise in ${y}_{\text{CO}}$.

#### Optimization of the hydrogen production process

The design expert software optimization tool was used to optimize the combined SR and PO processes. Therefore, to generate hydrogen-rich fuel cells efficiently, it is essential to optimize operating conditions to maximize hydrogen production and minimize CO emissions. Numerical optimization allows you to choose the preferred value for every input and response variable. In this case, the input adjustments that can be chosen are the in range, maximum, minimum, target, none (for responses), and setting an optimal response value for a specific set of conditions. In this research, the goal for all independent parameters (i.e., O/C, S/C, and T) was 'in range', and for responses, ${\text{y}}_{{\text{H}}_{2}}$ was set to'maximize' and ${\text{y}}_{\text{CO}}$ to'minimize'. The optimum condition with the greatest desirability is reported in Table 8.

**Table 8:** Hydrogen production optimization results using RSM.

Parameters	Goal	Lower limit	Upper limit	Optimal value
Oxygen-to-carbon ratio (O/C)	In range	0	2.8	0.4
Steam-to-carbon ratio(S/C)	In range	0.25	4	2.5
Temperature (T (°C) )	In range	100	600	250
Hydrogen mole fraction (${\text{y}}_{{{\text{H}}_{2} }}$)	Maximize	0.007	0.7246	0.414
Carbon monoxide mole fraction (${\text{y}}_{{{\text{CO}}}}$)	Minimize	0	0.3333	0.006

#### Effect of process parameters on ${\mathbf{y}}_{{\mathbf{H}}_{2}}$

**Figure 7:**
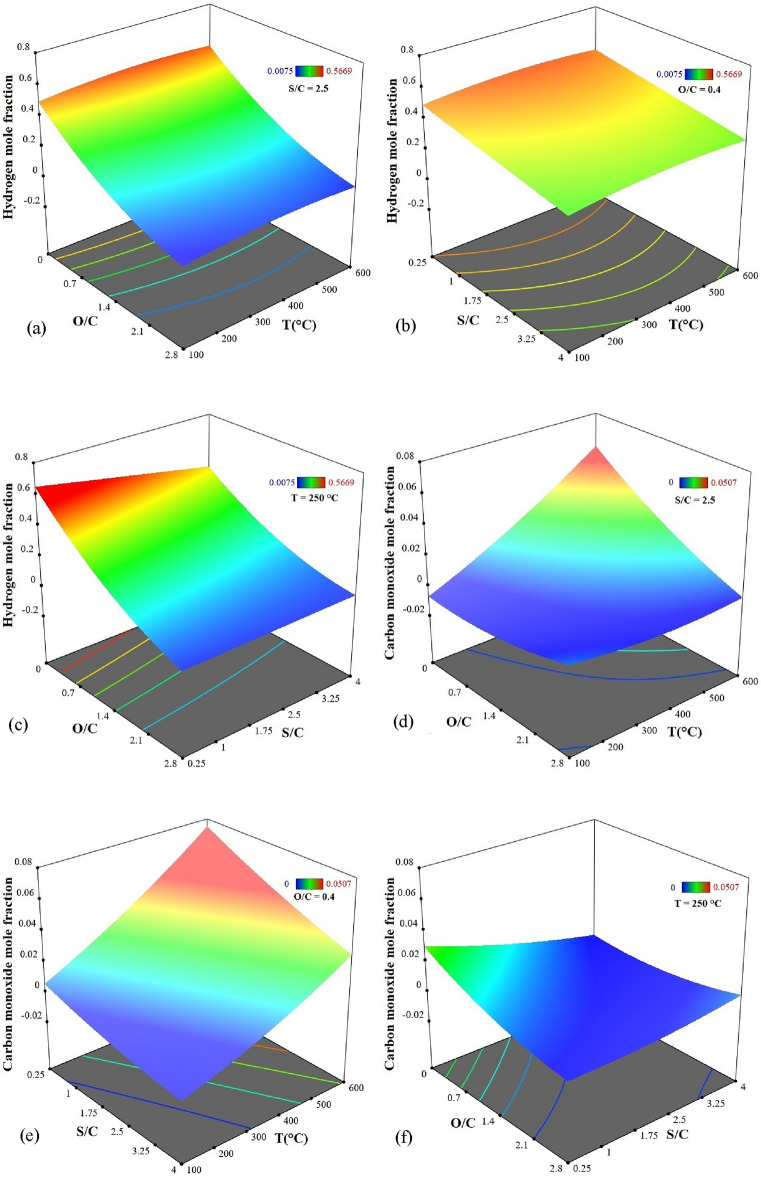
The 3D response surface plots showing simultaneous effects of (**a**) O/C and T on $y_{{H_{2} }}$, (**b**) S/C and T on $y_{{H_{2} }}$, (**c**) O/C and S/C on $y_{{H_{2} }}$, (**d**) O/C and T on $y_{CO}$, (**e**) S/C and T on $y_{CO}$ (**f**) O/C and S/C on $y_{CO}$.

The optimal condition in Table 8 was used to design three-dimensional (3D) response surface graphs. Figure 7 depicts the assessment of the interactions between independent variables and their impact on the responses. Figure 7a indicates that pure SR (O/C = 0) conditions maximize ${\text{y}}_{{\text{H}}_{2}}$. O/C significantly affects hydrogen content owing to fuel oxidation and nitrogen dilution. Increasing O/C beyond 0.50 adds more oxygen than is required for partial oxidation, triggering full oxidation^18^. The temperature has a slight impact on the hydrogen concentration; it increases gradually up to 300 °C and then gradually decreases as the temperature rises to 600 °C because of the WGSR equilibrium. The 3D surface in Fig. 7b shows that while the temperature has a minor impact on ${\text{y}}_{{\text{H}}_{2}}$, an increase in S/C leads to a decrease in ${\text{y}}_{{\text{H}}_{2}}$, primarily due to steam dilution. Compared to the effects of steam dilution, this shift in the WGSR equilibrium toward hydrogen and CO_2_ is negligible. According to the equilibrium of the WGSR, removing steam dilution increases hydrogen concentration^47^. A higher S/C suggests more steam in the system, and a higher O/C must lead to fewer CO emissions. However, as shown in Fig. 7c, due to steam dilution and fuel oxidation, the increases in O/C and S/C both result in a decrease in the end subscript. So, it is essential to use optimal values to minimize CO emissions while producing a considerable amount of hydrogen.

#### Effect of process parameters on ${\mathbf{y}}_{\mathbf{C}\mathbf{O}}$

As shown in Fig. 7d, the amount of CO increases with rising temperature owing to the WGSR equilibrium and reduces with increasing O/C due to the higher fraction of DME that is completely oxidized^12^. The maximum CO content occurs at O/C = 0 and the temperature of 600 °C. Figure 7e shows that as the S/C increases and the temperature decreases, ${\text{y}}_{\text{CO}}$ decreases as well. By increasing the S/C, the equilibrium is shifted towards the production of CO_2_ and hydrogen through the WGSR. As a result, the quantity of CO is reduced^22^. Figure 7f shows that increasing both O/C and S/C reduces CO due to more DME oxidization and shifts the WGSR equilibrium to CO_2_ and hydrogen production.

### ANN results

#### Optimizing the configuration of MLP and RBF networks

The optimum network configuration is set by trial and error by adjusting factors that impact the learning process, such as the number of neurons, training function, and activation function. Overtraining occurs when the neural network performs well exclusively on the training data but performs poorly on other domains, leading to poor precision and adaptability. It is important to reduce network running time and evaluate the model's performance using data that was not utilized for training in order to prevent overtraining.

##### MLP Training

**Figure 8:**
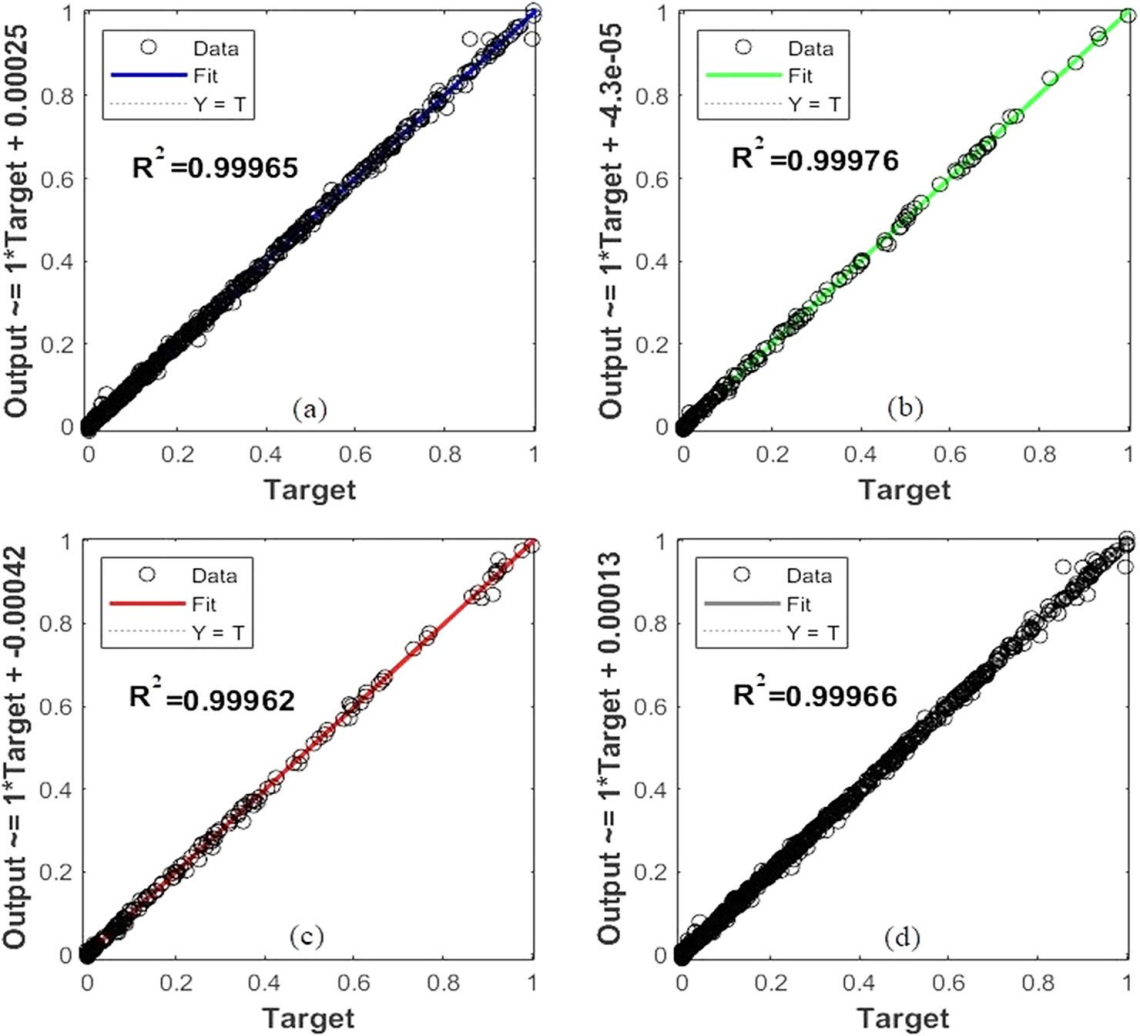
MLP network regression status of (a) training data, (b) validation data, (c) test data, and (d) all data for $y_{{H_{2} }}$ and $y_{CO}$.

**Figure 9:**
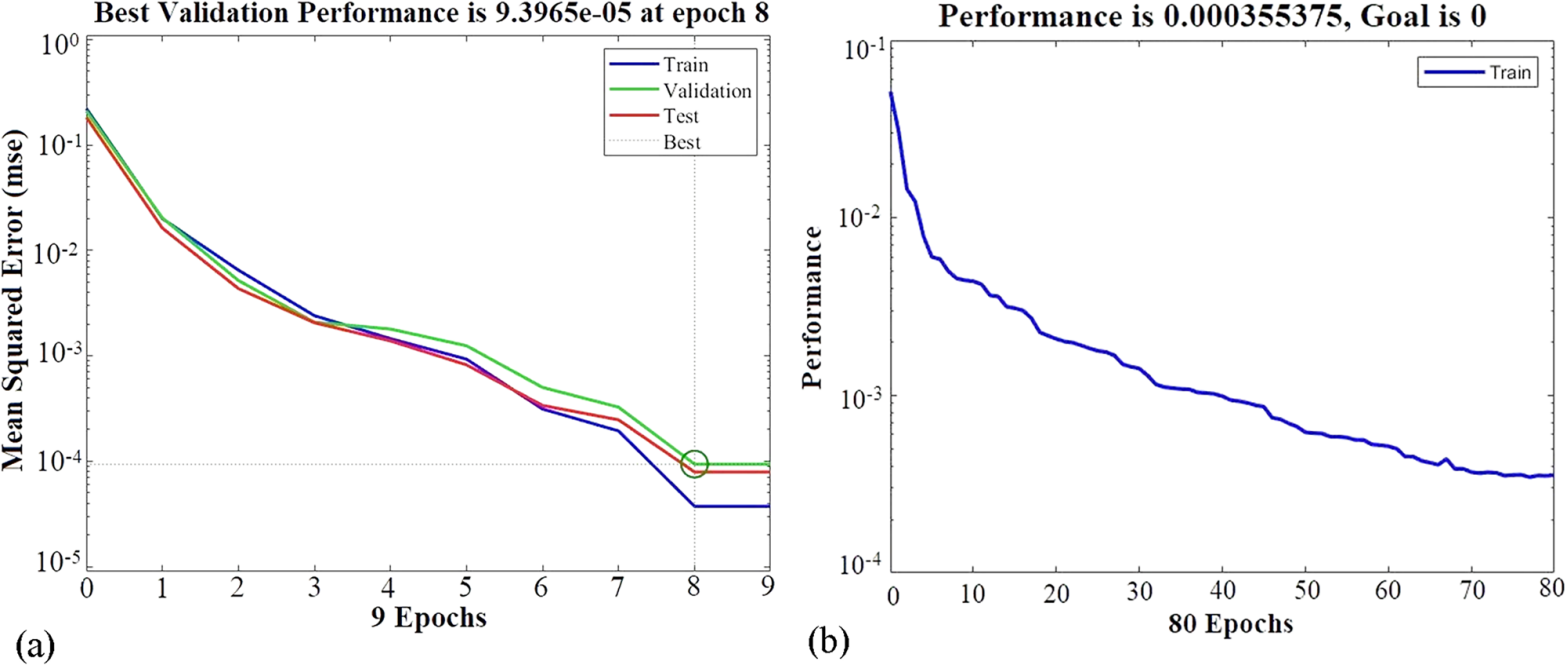
The MSE values during ANN training for (a) the MLP model and (b) the RBF model.

The normalized data were split into three groups in order to train the MLP network: 70% of the data were randomly assigned to network training, 15% to validation, and 15% to network testing. To determine the optimal algorithm for the MLP network, three distinct training algorithms, including Levenberg–Marquardt (trainlm), Bayesian Regularization (trainbr), and Scaled Conjugate Gradient (trainscg), were examined. In order to get optimal outcomes on the test data, the configuration of the MLP network was carefully evaluated. This included the training function, the hidden layer sizes and neurons in each layer, the activation function used in each layer, and the number of epochs. Among the distinct activation functions that have been applied in this research, the sigmoid function (tansig) and linear function (purelin) have been chosen as the activation functions for the neurons in the hidden and output layers, respectively, as shown in Table 9. The outcome of several MLP configurations is shown in Table 10. The trainlm function was selected as the best approach to train the network as it performs better than all evaluated setups on all performance metrics, such as MSE, MAE, MAPE, *R*^2^, and the number of epochs. An increase in the number of neurons and the size of the hidden layers led to a decrease in the MSE value. The MSE value was not significantly affected by increasing the number of hidden layers beyond three; therefore, in order to avoid an extended network running time, three hidden layers were considered sufficient. The optimal configuration of the MLP network was obtained with three hidden layer sizes and neuron numbers of 15, 10, and 5, respectively. The lowest MSE values and the highest *R*^2^ were achieved. The best MSE values for ${\text{y}}_{{\text{H}}_{2}}$ and ${\text{y}}_{\text{CO}}$ were 3.9525e−05 and 4.8876e−05, and the best *R*^2^ values for ${\text{y}}_{{\text{H}}_{2}}$ and ${\text{y}}_{\text{CO}}$ were 0.99973 and 0.99916, respectively. The details of the optimal MLP network weight matrix and bias values can be found in **Error! Reference source not found.** of supplementary. Figure 8 illustrates the regression state of the training data, validation data, testing data, and the whole dataset. The network performance throughout the training process is shown in Fig. 9a. The MLP network attained the ideal MSE validation results for nine epochs.

**Table 9:** Mathematical and graphical representations of activation functions.

Mathematical algorithm function	Graph
$$a = purelin\left( n \right) = n$$	
$$a = tansig\left( n \right) = \frac{2}{{1 + e^{ - 2n} }} - 1$$

**Table 10:** The results of several evaluated MLP training algorithms.

Train function	Hidden layer size	MSE Train		MSE Test		22MAE Train		MAE Test		MAPE Train		MAPE Test		*R*^2^		Epoch
		$y_{{H_{2} }}$	$y_{CO}$	$y_{{H_{2} }}$	$y_{CO}$	$y_{{H_{2} }}$	$y_{CO}$	$y_{{H_{2} }}$	$y_{CO}$	$y_{{H_{2} }}$	$y_{CO}$	$y_{{H_{2} }}$	$y_{CO}$	$y_{{H_{2} }}$	$y_{CO}$	
Trainlm	^10^	0.0011	0.0023	0.0016	0.0032	0.0231	0.0241	0.0278	0.0316	31.35	81.28	49.99	86.93	0.9925	0.9599	8
Trainlm	[10 5]	0.0009	0.0005	0.0016	0.0026	0.0191	0.0127	0.0268	0.0243	14.08	73.06	19.84	85.70	0.9944	0.9915	10
**Trainlm**	**[15 10 5]**	**0.00004**	**0.00005**	**0.0001**	**0.0018**	**0.0041**	**0.0036**	**0.0056**	**0.0203**	**4.53**	**19.54**	**7.08**	**22.80**	**0.9997**	**0.9992**	**9**
Trainlm	[20 15 10 5]	0.0003	2.67–4	0.0004	0.0021	0.0117	0.0077	0.0158	0.0229	14.65	103.82	24.71	76.31	0.9981	0.9955	12
Trainbr	^10^	0.0011	0.0014	0.0015	0.0037	0.0231	0.0209	0.0275	0.0344	39.65	107.84	75.88	142.28	0.9923	0.9763	16
Trainbr	[10 5]	0.0006	0.0010	0.0014	0.0043	0.0118	0.0121	0.0162	0.0215	15.05	61.43	48.42	82.85	0.9957	0.9825	18
Trainbr	[15 10 5]	0.0001	0.0003	0.0002	0.0020	0.0059	0.0081	0.0082	0.0216	7.99	28.54	9.27	45.28	0.9993	0.9949	19
Trainbr	[20 15 10 5]	0.0001	0.0002	0.0002	0.0026	0.0064	0.0061	0.0072	0.0237	8.05	31.18	8.96	48.51	0.9993	0.9972	32
Trainscg	^10^	0.0055	0.0070	0.0060	0.0141	0.0539	0.0527	0.0606	0.0682	30.76	97.16	40.07	120.33	0.9622	0.8724	45
Trainscg	[10 5]	0.0012	0.0014	0.0012	0.0034	0.0267	0.0210	0.0279	0.0320	21.65	62.27	25.38	85.81	0.9920	0.9756	90
Trainscg	[15 10 5]	0.0007	0.0008	0.0010	0.0024	0.0199	0.0160	0.0234	0.0235	20.66	48.61	24.45	73.46	0.9956	0.9862	125
Trainscg	[20 15 10 5]	0.0004	0.0004	0.0011	0.0026	0.0154	0.0125	0.0205	0.0272	14.37	34.41	21.24	81.25	0.9970	0.9933	140

Bold row in Table 10, represent the optimum structure of MLP.

##### RBF Training

As mentioned before, every neural network consists of input, hidden, and output layers. However, the RBF neural network is distinguished by its singular hidden layer and the output of a linear function. This feedforward network variation could require a greater number of hidden-layer neurons than others. After normalizing the data, the impact of different numbers of neurons (ranging from 1 to 591) and spread values on the training state of the network was studied. By setting the spread value less than one and the number of neurons to the maximum value of 591, the MSE value of the training stage was reduced significantly (almost to 0). However, this led to overtraining of the network, and the MSE value of the test data was not acceptable. Thus, the Gaussian function with a spread value of 2.5 and 80 epochs was determined to be the optimal RBF network configuration by trial and error. Where the MSE values for ${\text{y}}_{{\text{H}}_{2}}$ and ${\text{y}}_{\text{CO}}$ were 1.3611e−04 and 5.7466e−04, and the *R*^2^ values for ${\text{y}}_{{\text{H}}_{2}}$ and ${\text{y}}_{\text{CO}}$ were 0.99908 and 0.99011, respectively. Figure 9b shows the learning curve based on the optimal topology of the RBF network.

#### 3D response surfaces

**Figure 10:**
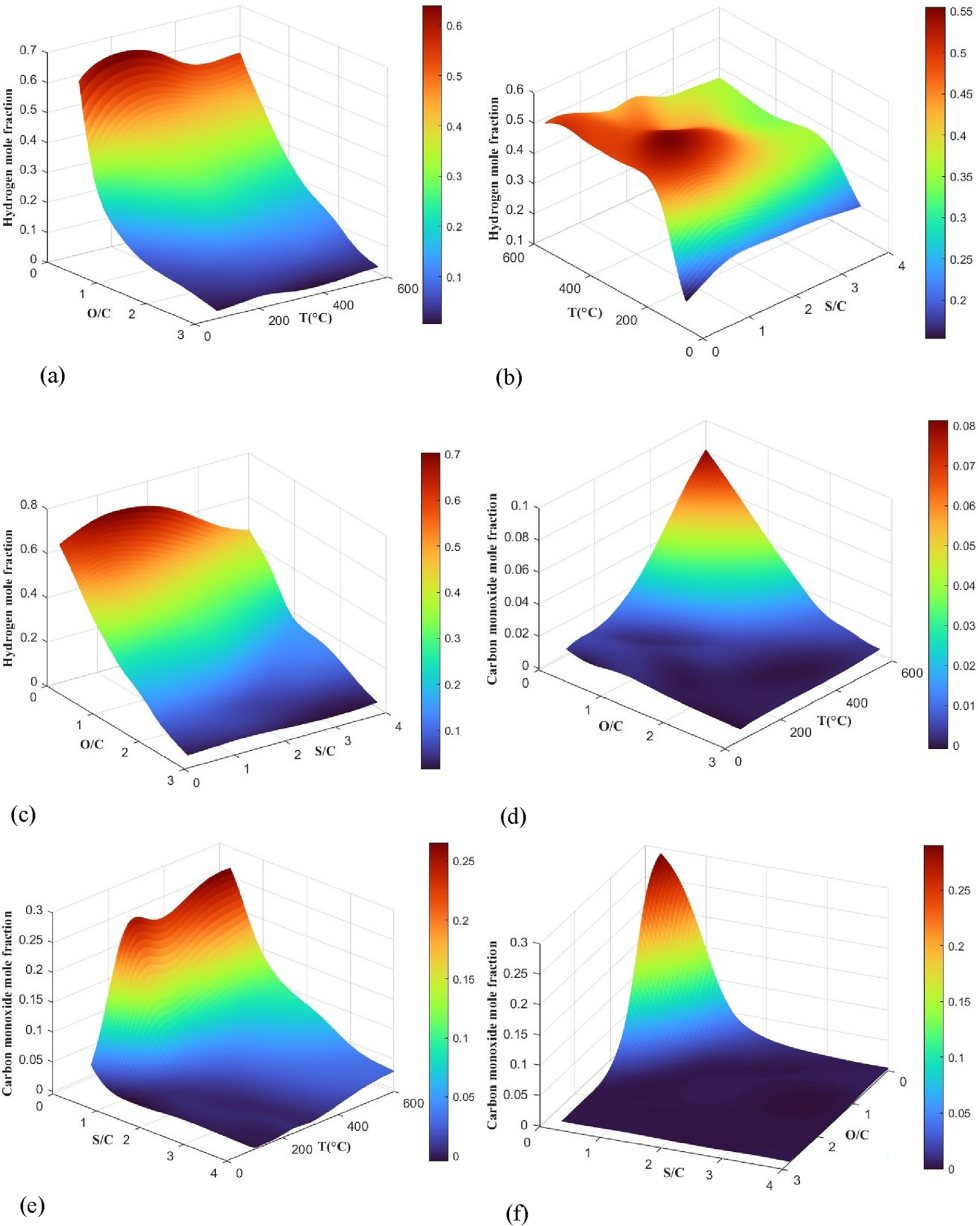
3D response surface plots based on ANN by MLP model (a) O/C and T on $y_{{H_{2} }}$, (b) S/C and T on $y_{{H_{2} }}$, (c) O/C and S/C on $y_{{H_{2} }}$, (d) O/C and T on $y_{CO}$, (e) S/C and T on $y_{CO}$, (f) O/C and S/C on $y_{CO}$.

To assess the impact of process parameters on both responses using MLP and RBF networks, data on two parameters as variables and another parameter with a constant value were given into trained networks as inputs, and ${\text{y}}_{{\text{H}}_{2}}$ and ${\text{y}}_{\text{CO}}$ values were computed. The 3D plots in Fig. 10(a–f) show the response surface of the best ANN configuration model under various conditions for ${\text{y}}_{{\text{H}}_{2}}$ and ${\text{y}}_{\text{CO}}$. To ensure a fair comparison, the constant variables in each plot are the same as the optimal condition in RSM. Based on the results, it looks like the factors that affect both ${\text{y}}_{{\text{H}}_{2}}$ and ${\text{y}}_{\text{CO}}$ show the same behavior and trends as RSM.

### Comparison of RSM and ANN results

Several rows of randomly chosen thermodynamic data were fed into the RSM model, MLP, and RBF networks in order to compare the outcomes of the trained networks with the model acquired by the RSM approach. The reliability of the models and networks was assessed by comparing the predicted values of ${\text{y}}_{{\text{H}}_{2}}$ and ${\text{y}}_{\text{CO}}$ with the actual data and computing the average absolute relative deviation (%AARD). The comparative results between the RSM model and the ANNs are shown in Table 11. Given their developed predictability, the neural networks delivered lower %AARD values than the RSM model. Furthermore, it was determined that the MLP network outperformed the RBF network in terms of accuracy in predicting the output parameters.

**Table 11:** Comparison results for several case studies conducted by MLP, RBF, and RSM.

Run	(O/C)	(S/C)	T (°C)	RSM				MLP				RBF			
				$y_{{H_{2} }}$		$y_{CO}$		$y_{{H_{2} }}$		$y_{CO}$		$y_{{H_{2} }}$		$y_{CO}$	
				Y_RSM_	AARD	Y_RSM_	AARD	Y_MLP_	AARD	Y_MLP_	AARD	Y_RBF_	AARD	Y_RBF_	AARD
1	0.40	2.5	300	0.42	2.02	0.01	24.25	0.44	0.36	0.01	2.36	0.44	0.06	0.00	45.81
2	2.00	0.75	500	0.13	1.82	0.02	0.55	0.13	0.53	0.02	0.28	0.13	0.79	0.02	2.29
3	0	1.25	200	0.59	9.45	0.01	40.20	0.72	0.32	0.07	2.53	0.73	0.15	0.07	3.37
4	0.80	1.5	500	0.36	1.21	0.03	13.56	0.35	0.05	0.05	0.27	0.35	0.70	0.04	5.68
5	1.20	2.5	400	0.24	0.48	0.01	15.23	0.25	1.29	0.01	10.51	0.24	0.56	0.02	55.88
6	1.60	1.5	500	0.19	1.54	0.02	1.50	0.18	0.82	0.02	2.17	0.18	1.19	0.02	1.81
7	1.60	3	600	0.14	2.74	0.02	6.08	0.15	0.39	0.01	2.75	0.16	4.13	0.02	11.93
8	0.40	1.5	300	0.47	4.69	0.02	9.12	0.52	0.13	0.02	2.58	0.51	0.78	0.03	14.12
9	2.40	2.25	400	0.07	2.03	0.00	29.19	0.06	2.16	0.00	2.63	0.07	3.96	0.00	6.25
10	0.80	0.5	300	0.40	1.25	0.02	34.92	0.41	0.28	0.07	0.23	0.42	0.61	0.08	11.37

## Conclusion

The rising need for analysis and process optimization, in addition to the increased accessibility of statistical software and enhanced computational capabilities, has resulted in the extensive use of Response Surface Methodology (RSM) and Artificial Neural Network (ANN) modeling tools. This research focused on analyzing the hydrogen production process factors, including the oxygen-to-carbon ratio, steam-to-carbon ratio, and temperature. The goal was to predict the responses of ${\text{y}}_{{\text{H}}_{2}}$ and ${\text{y}}_{\text{CO}}$ using RSM and ANN modeling methods. RSM modeling was used to assess the interaction effects of the elements under consideration. The results achieved from both modeling approaches exhibited a consistent and precise prediction of the thermodynamic data. In addition, the RSM used the quadratic model to establish two correlations for the responses. The optimal operating conditions for maximizing hydrogen generation and minimizing CO emissions in the combined processes of dimethyl ether steam reforming and partial oxidation were obtained at O/C of 0.4, S/C of 2.5, and temperature of 250 °C. The MLP and RBF algorithms were assessed for modeling the thermodynamic data on hydrogen generation, and their outcomes were almost identical upon comparison. After comparing the MLP algorithms, it was concluded that the Trainlm algorithm showed the greatest promise. This approach used the tansig activation function in the hidden layers and the purelin activation function in the output layer. The MLP network demonstrated superior performance in terms of mean squared error (MSE) with values of 3.9525e−05 and 4.8876e−05 for hydrogen mole fraction and carbon monoxide mole fraction, respectively, after nine epochs. On the other hand, the RBF network achieved MSE values of 1.3611e−04 and 5.7466e−04 for hydrogen mole fraction and carbon monoxide mole fraction, respectively, after 80 epochs. The hydrogen production process model, which used both ANN and RSM, achieved mean *R*^2^ values of 0.9994 and 0.9771, respectively. Overall, the ANN method outperformed the RSM in modeling the hydrogen production process.

### Supplementary Information

Supplementary Information.

## Data Availability

The data used and analyzed during the current work is available from the corresponding author upon reasonable request.

## List of symbols

*b*: Bias (–)
*C*_*i*_: Center point (–)
*f*: Function
Φ_*i*_: Gaussian function
*i*: Number of neurons in the hidden layer
*N*: Number of data sets for training (–)
*n*: Neurons
*R*^2^: Correlation coefficient (%)
*W*: Weight factor (–)
*W*_*ij*_: Weight-related to each hidden neuron (–)
*x*_*i*_: Input examples (attributes)
*Y*: Output vector (–)
*y*_*j*_: Target output
$$y_{{H_{2} }}$$: H_2_ mole fraction
$y_{CO}$: CO mole fraction

## Greek symbols

*α*: Constants in turbulence models (–)
*β*: Interaction coefficient
$\gamma_{jk}$: Neuron j’s output from k’s layer
*σ*_*i*_: Spread of Gaussian function (−

## Abbreviations

AARD: Average absolute relative deviation (%)
ANN: Artificial neural networks
MAE: Mean absolute error
MAPE: Mean absolute percentage error
MSE: Mean square error
S/C: Steam/carbon
O/C: Oxygen/carbon

## Terminology

Neurons: Neurons are the basic units of the large neural network
Bias: Bias is a constant that helps the model fit the given data best
Activation function: The activation function is a mathematical function between the input feeding current neuron and its output going to the next layer
Weight: Represents the importance and strengths of the feature/input to the neurons
Epoch: In the training process, the inputs enter each training step and give an output compared with the target to calculate an error. This process calculates and modifies weights and biases in each epoch
