# Supplementary material for: Evaluation of hydrogen production via steam reforming and partial oxidation of dimethyl ether using response surface methodology and artificial neural network
Source: Sci Rep. 2024 Jul 6;14:15570. doi: 10.1038/s41598-024-66402-5 (PMC11227547; doi:10.1038/s41598-024-66402-5)
Supplement: Supplementary file 1 — Supplementary Information. [file 41598_2024_66402_MOESM1_ESM.docx]

**Supplementary**

Table S1. Weight matrix and bias values of the optimal MLP network.

| Neurons |  | 1 | 2 | 3 | 4 | 5 | 6 | 7 | 8 | 9 | 10 | 11 | 12 | 13 | | 14 | 15 |
| --- | --- | --- | --- | --- | --- | --- | --- | --- | --- | --- | --- | --- | --- | --- | --- | --- | --- |
| First hidden layer | w_i_ | 2.32 | -1.68 | -2.54 | 0.01 | -2.03 | -0.90 | 1.69 | 2.16 | 2.44 | -1.73 | 0.60 | -1.49 | 0.27 | 3.76 | | -0.86 |
|  |  | 2.03 | -2.39 | -1.28 | -0.06 | -0.74 | -2.46 | -0.22 | -2.60 | -0.51 | -1.81 | 0.38 | -0.65 | 2.92 | 0.04 | | -1.00 |
|  |  | 0.85 | -0.91 | -0.85 | -2.86 | -1.53 | -1.89 | 0.14 | -0.41 | -2.23 | -1.34 | -1.44 | -0.99 | -0.41 | -0.23 | | 1.92 |
|  | b | -3.63 | 3.23 | 1.93 | -2.61 | 0.99 | 1.19 | -1.76 | -0.19 | -0.24 | -3.22 | 0.37 | -0.68 | 3.77 | 3.54 | | -2.83 |
| Second hidden layer | w_i_ | -0.22 | -0.19 | -0.60 | -0.65 | 0.00 | -0.27 | -0.58 | 0.80 | 0.18 | 0.93 |  |  |  |  | |  |
|  |  | 0.13 | -0.69 | -0.17 | -0.45 | 0.05 | 0.25 | 0.72 | 0.14 | 0.03 | -0.63 |  |  |  |  | |  |
|  |  | -0.15 | 0.55 | -0.70 | -0.97 | -0.33 | 0.25 | -0.35 | -0.37 | 0.39 | -0.20 |  |  |  |  | |  |
|  |  | -0.38 | 0.33 | 0.64 | 1.20 | 0.90 | 0.58 | 0.53 | -0.30 | -0.82 | -0.71 |  |  |  |  | |  |
|  |  | 0.04 | -1.00 | 0.52 | -0.49 | -0.02 | -0.47 | -0.47 | 0.04 | 0.09 | 0.26 |  |  |  |  | |  |
|  |  | 0.04 | 0.07 | 0.15 | -0.44 | 0.13 | -0.06 | 0.05 | -0.08 | 0.01 | 0.24 |  |  |  |  | |  |
|  |  | -0.34 | 0.31 | 0.24 | -0.10 | 1.14 | 0.13 | 0.23 | 0.13 | -0.51 | -0.62 |  |  |  |  | |  |
|  |  | 0.26 | -0.66 | 0.72 | 0.52 | -0.01 | -0.07 | 0.07 | -0.36 | 0.06 | -0.46 |  |  |  |  | |  |
|  |  | -0.01 | -0.65 | 0.18 | 0.57 | -0.03 | -0.10 | 0.02 | 0.23 | 0.36 | -0.81 |  |  |  |  | |  |
|  |  | 1.01 | -0.16 | -0.75 | 0.16 | 0.04 | -0.56 | -0.56 | 0.86 | -0.32 | -0.31 |  |  |  |  | |  |
|  |  | -1.14 | -0.25 | 0.80 | 1.20 | 0.21 | 0.78 | 0.34 | -0.75 | 0.26 | -0.11 |  |  |  |  | |  |
|  |  | 0.49 | -0.08 | -0.22 | -1.06 | -0.07 | -0.19 | -0.57 | 0.60 | -1.30 | 0.83 |  |  |  |  | |  |
|  |  | -0.40 | 0.63 | 1.47 | 0.34 | -0.22 | -0.57 | -0.07 | -0.97 | -1.22 | -0.17 |  |  |  |  | |  |
|  |  | 1.15 | -0.73 | 0.27 | -0.41 | 1.21 | -0.11 | -0.08 | 0.01 | 0.59 | -0.84 |  |  |  |  | |  |
|  |  | 0.31 | 0.00 | -0.12 | -0.20 | 0.49 | -0.58 | -0.16 | 0.21 | -0.14 | -0.13 |  |  |  |  | |  |
|  | b | -1.31 | 0.17 | 0.87 | 0.62 | 0.49 | 0.08 | -0.18 | 1.11 | 1.44 | 1.36 |  |  |  |  | |  |
| Third hidden layer | w_i_ | -0.39 | -0.19 | -0.42 | -0.42 | -0.03 |  |  |  |  |  |  |  |  |  | |  |
|  |  | 0.67 | -0.93 | 0.06 | -0.10 | 0.31 |  |  |  |  |  |  |  |  |  | |  |
|  |  | 1.01 | 0.09 | 1.20 | -0.13 | -0.61 |  |  |  |  |  |  |  |  |  | |  |
|  |  | 0.55 | 0.78 | 1.12 | -0.03 | -0.20 |  |  |  |  |  |  |  |  |  | |  |
|  |  | -0.32 | 0.85 | 0.10 | -0.69 | -0.72 |  |  |  |  |  |  |  |  |  | |  |
|  |  | 0.84 | 0.07 | 0.43 | 0.12 | -1.16 |  |  |  |  |  |  |  |  |  | |  |
|  |  | -0.40 | -0.20 | 0.59 | -0.88 | 0.05 |  |  |  |  |  |  |  |  |  | |  |
|  |  | -1.38 | 0.81 | -0.53 | -1.46 | -0.73 |  |  |  |  |  |  |  |  |  | |  |
|  |  | -0.33 | 0.73 | 0.07 | -0.51 | -1.63 |  |  |  |  |  |  |  |  |  | |  |
|  |  | -0.22 | 0.77 | 0.27 | 0.03 | 0.23 |  |  |  |  |  |  |  |  |  | |  |
|  | b | 2.07 | -0.51 | 0.27 | -2.02 | 0.41 |  |  |  |  |  |  |  |  |  | |  |
| Output layer | w_l_ | 0.47 | 1.08 |  |  |  |  |  |  |  |  |  |  |  |  | |  |
|  |  | -0.08 | -0.04 |  |  |  |  |  |  |  |  |  |  |  |  | |  |
|  |  | -0.678 | -1.173 |  |  |  |  |  |  |  |  |  |  |  |  | |  |
|  |  | 1.075 | -0.173 |  |  |  |  |  |  |  |  |  |  |  |  | |  |
|  |  | 0.258 | 0.04 |  |  |  |  |  |  |  |  |  |  |  |  | |  |
|  | b | 0.576 | -1.003 |  |  |  |  |  |  |  |  |  |  |  |  | |  |
| w_i_: network weights between input and hidden layer neurons.  w_l_: network weights between hidden and output layer neurons. | | | | | | | | | | | | | | | | | |

Table S2: Data points that were collected from the literature.

| No. | Operating Conditions | | | Mole Fraction | |
| --- | --- | --- | --- | --- | --- |
|  | O/C | S/C | T (°C) | H_2_ | CO |
| 1 | 0 | 0.25 | 100 | 0.4485 | 0.0018 |
| 2 | 0 | 0.25 | 200 | 0.5475 | 0.2247 |
| 3 | 0 | 0.25 | 300 | 0.5714 | 0.2857 |
| 4 | 0 | 0.25 | 400 | 0.5714 | 0.2857 |
| 5 | 0 | 0.25 | 500 | 0.5714 | 0.2857 |
| 6 | 0 | 0.25 | 600 | 0.5714 | 0.2857 |
| 7 | 0.40 | 0.25 | 100 | 0.0943 | 0.0072 |
| 8 | 0.40 | 0.25 | 200 | 0.3992 | 0.2084 |
| 9 | 0.40 | 0.25 | 300 | 0.4871 | 0.2472 |
| 10 | 0.40 | 0.25 | 400 | 0.4916 | 0.2492 |
| 11 | 0.40 | 0.25 | 500 | 0.4877 | 0.2546 |
| 12 | 0.40 | 0.25 | 600 | 0.482 | 0.2603 |
| 13 | 0.80 | 0.25 | 100 | 0.0802 | 0.0059 |
| 14 | 0.80 | 0.25 | 200 | 0.331 | 0.1117 |
| 15 | 0.80 | 0.25 | 300 | 0.3907 | 0.1167 |
| 16 | 0.80 | 0.25 | 400 | 0.3842 | 0.1309 |
| 17 | 0.80 | 0.25 | 500 | 0.3685 | 0.148 |
| 18 | 0.80 | 0.25 | 600 | 0.3534 | 0.1635 |
| 19 | 1.20 | 0.25 | 100 | 0.0745 | 0.0027 |
| 20 | 1.20 | 0.25 | 200 | 0.2708 | 0.021 |
| 21 | 1.20 | 0.25 | 300 | 0.3102 | 0.0403 |
| 22 | 1.20 | 0.25 | 400 | 0.2952 | 0.0627 |
| 23 | 1.20 | 0.25 | 500 | 0.2752 | 0.0839 |
| 24 | 1.20 | 0.25 | 600 | 0.2574 | 0.102 |
| 25 | 1.60 | 0.25 | 100 | 0.0529 | 0 |
| 26 | 1.60 | 0.25 | 200 | 0.1924 | 0.0022 |
| 27 | 1.60 | 0.25 | 300 | 0.2243 | 0.0122 |
| 28 | 1.60 | 0.25 | 400 | 0.2135 | 0.0284 |
| 29 | 1.60 | 0.25 | 500 | 0.1969 | 0.0459 |
| 30 | 1.60 | 0.25 | 600 | 0.1812 | 0.0618 |
| 31 | 2.00 | 0.25 | 100 | 0.0355 | 0 |
| 32 | 2.00 | 0.25 | 200 | 0.1245 | 0.0007 |
| 33 | 2.00 | 0.25 | 300 | 0.1453 | 0.0044 |
| 34 | 2.00 | 0.25 | 400 | 0.1403 | 0.0126 |
| 35 | 2.00 | 0.25 | 500 | 0.1298 | 0.0236 |
| 36 | 2.00 | 0.25 | 600 | 0.1186 | 0.035 |
| 37 | 2.40 | 0.25 | 100 | 0.0216 | 0 |
| 38 | 2.40 | 0.25 | 200 | 0.0695 | 0.0003 |
| 39 | 2.40 | 0.25 | 300 | 0.0793 | 0.0016 |
| 40 | 2.40 | 0.25 | 400 | 0.0772 | 0.0051 |
| 41 | 2.40 | 0.25 | 500 | 0.072 | 0.0105 |
| 42 | 2.40 | 0.25 | 600 | 0.0657 | 0.0169 |
| 43 | 2.80 | 0.25 | 100 | 0.0086 | 0 |
| 44 | 2.80 | 0.25 | 200 | 0.0224 | 0.0001 |
| 45 | 2.80 | 0.25 | 300 | 0.0243 | 0.0004 |
| 46 | 2.80 | 0.25 | 400 | 0.0237 | 0.0012 |
| 47 | 2.80 | 0.25 | 500 | 0.0222 | 0.0027 |
| 48 | 2.80 | 0.25 | 600 | 0.0203 | 0.0046 |
| 49 | 0 | 0.5 | 100 | 0.5724 | 0.0013 |
| 50 | 0 | 0.5 | 200 | 0.638 | 0.1966 |
| 51 | 0 | 0.5 | 300 | 0.6664 | 0.3323 |
| 52 | 0 | 0.5 | 400 | 0.6667 | 0.3333 |
| 53 | 0 | 0.5 | 500 | 0.6667 | 0.3333 |
| 54 | 0 | 0.5 | 600 | 0.6667 | 0.3333 |
| 55 | 0.40 | 0.5 | 100 | 0.1302 | 0.0046 |
| 56 | 0.40 | 0.5 | 200 | 0.4461 | 0.1537 |
| 57 | 0.40 | 0.5 | 300 | 0.5148 | 0.1691 |
| 58 | 0.40 | 0.5 | 400 | 0.5111 | 0.1804 |
| 59 | 0.40 | 0.5 | 500 | 0.4971 | 0.1953 |
| 60 | 0.40 | 0.5 | 600 | 0.4835 | 0.2093 |
| 61 | 0.80 | 0.5 | 100 | 0.0998 | 0.0037 |
| 62 | 0.80 | 0.5 | 200 | 0.3577 | 0.0528 |
| 63 | 0.80 | 0.5 | 300 | 0.41 | 0.0679 |
| 64 | 0.80 | 0.5 | 400 | 0.3954 | 0.091 |
| 65 | 0.80 | 0.5 | 500 | 0.3741 | 0.1137 |
| 66 | 0.80 | 0.5 | 600 | 0.3551 | 0.133 |
| 67 | 1.20 | 0.5 | 100 | 0.0759 | 0.0001 |
| 68 | 1.20 | 0.5 | 200 | 0.2705 | 0.005 |
| 69 | 1.20 | 0.5 | 300 | 0.3119 | 0.0216 |
| 70 | 1.20 | 0.5 | 400 | 0.2972 | 0.0436 |
| 71 | 1.20 | 0.5 | 500 | 0.2768 | 0.0652 |
| 72 | 1.20 | 0.5 | 600 | 0.2583 | 0.084 |
| 73 | 1.60 | 0.5 | 100 | 0.0518 | 0 |
| 74 | 1.60 | 0.5 | 200 | 0.1862 | 0.0013 |
| 75 | 1.60 | 0.5 | 300 | 0.2188 | 0.0079 |
| 76 | 1.60 | 0.5 | 400 | 0.211 | 0.0208 |
| 77 | 1.60 | 0.5 | 500 | 0.1962 | 0.0364 |
| 78 | 1.60 | 0.5 | 600 | 0.1814 | 0.0514 |
| 79 | 2.00 | 0.5 | 100 | 0.0349 | 0 |
| 80 | 2.00 | 0.5 | 200 | 0.1207 | 0.0005 |
| 81 | 2.00 | 0.5 | 300 | 0.141 | 0.0033 |
| 82 | 2.00 | 0.5 | 400 | 0.1374 | 0.0098 |
| 83 | 2.00 | 0.5 | 500 | 0.1285 | 0.0192 |
| 84 | 2.00 | 0.5 | 600 | 0.1184 | 0.0295 |
| 85 | 2.40 | 0.5 | 100 | 0.0213 | 0 |
| 86 | 2.40 | 0.5 | 200 | 0.0675 | 0.0002 |
| 87 | 2.40 | 0.5 | 300 | 0.077 | 0.0013 |
| 88 | 2.40 | 0.5 | 400 | 0.0754 | 0.0042 |
| 89 | 2.40 | 0.5 | 500 | 0.071 | 0.0088 |
| 90 | 2.40 | 0.5 | 600 | 0.0654 | 0.0144 |
| 91 | 2.80 | 0.5 | 100 | 0.0085 | 0 |
| 92 | 2.80 | 0.5 | 200 | 0.0218 | 0.0001 |
| 93 | 2.80 | 0.5 | 300 | 0.0236 | 0.0003 |
| 94 | 2.80 | 0.5 | 400 | 0.0231 | 0.001 |
| 95 | 2.80 | 0.5 | 500 | 0.0219 | 0.0023 |
| 96 | 2.80 | 0.5 | 600 | 0.0202 | 0.004 |
| 97 | 0 | 0.75 | 100 | 0.6277 | 0.001 |
| 98 | 0 | 0.75 | 200 | 0.6819 | 0.1588 |
| 99 | 0 | 0.75 | 300 | 0.6872 | 0.2359 |
| 100 | 0 | 0.75 | 400 | 0.6782 | 0.2449 |
| 101 | 0 | 0.75 | 500 | 0.6665 | 0.2566 |
| 102 | 0 | 0.75 | 600 | 0.6556 | 0.2675 |
| 103 | 0.40 | 0.75 | 100 | 0.1674 | 0.003 |
| 104 | 0.40 | 0.75 | 200 | 0.4782 | 0.0939 |
| 105 | 0.40 | 0.75 | 300 | 0.5336 | 0.1064 |
| 106 | 0.40 | 0.75 | 400 | 0.5197 | 0.1281 |
| 107 | 0.40 | 0.75 | 500 | 0.4982 | 0.151 |
| 108 | 0.40 | 0.75 | 600 | 0.4789 | 0.1706 |
| 109 | 0.80 | 0.75 | 100 | 0.1143 | 0.0006 |
| 110 | 0.80 | 0.75 | 200 | 0.3687 | 0.0134 |
| 111 | 0.80 | 0.75 | 300 | 0.4149 | 0.0372 |
| 112 | 0.80 | 0.75 | 400 | 0.3968 | 0.0638 |
| 113 | 0.80 | 0.75 | 500 | 0.3733 | 0.0888 |
| 114 | 0.80 | 0.75 | 600 | 0.3526 | 0.1099 |
| 115 | 1.20 | 0.75 | 100 | 0.074 | 0 |
| 116 | 1.20 | 0.75 | 200 | 0.2615 | 0.0024 |
| 117 | 1.20 | 0.75 | 300 | 0.3051 | 0.0132 |
| 118 | 1.20 | 0.75 | 400 | 0.2936 | 0.0317 |
| 119 | 1.20 | 0.75 | 500 | 0.2746 | 0.0519 |
| 120 | 1.20 | 0.75 | 600 | 0.2565 | 0.0702 |
| 121 | 1.60 | 0.75 | 100 | 0.0507 | 0 |
| 122 | 1.60 | 0.75 | 200 | 0.1801 | 0.0009 |
| 123 | 1.60 | 0.75 | 300 | 0.2121 | 0.0057 |
| 124 | 1.60 | 0.75 | 400 | 0.2066 | 0.016 |
| 125 | 1.60 | 0.75 | 500 | 0.1937 | 0.0296 |
| 126 | 1.60 | 0.75 | 600 | 0.18 | 0.0435 |
| 127 | 2.00 | 0.75 | 100 | 0.0343 | 0 |
| 128 | 2.00 | 0.75 | 200 | 0.1171 | 0.0004 |
| 129 | 2.00 | 0.75 | 300 | 0.1366 | 0.0026 |
| 130 | 2.00 | 0.75 | 400 | 0.1341 | 0.0079 |
| 131 | 2.00 | 0.75 | 500 | 0.1265 | 0.016 |
| 132 | 2.00 | 0.75 | 600 | 0.1173 | 0.0253 |
| 133 | 2.40 | 0.75 | 100 | 0.0209 | 0 |
| 134 | 2.40 | 0.75 | 200 | 0.0656 | 0.0002 |
| 135 | 2.40 | 0.75 | 300 | 0.0747 | 0.0011 |
| 136 | 2.40 | 0.75 | 400 | 0.0735 | 0.0035 |
| 137 | 2.40 | 0.75 | 500 | 0.0697 | 0.0075 |
| 138 | 2.40 | 0.75 | 600 | 0.0648 | 0.0125 |
| 139 | 2.80 | 0.75 | 100 | 0.0083 | 0 |
| 140 | 2.80 | 0.75 | 200 | 0.0212 | 0 |
| 141 | 2.80 | 0.75 | 300 | 0.0229 | 0.0003 |
| 142 | 2.80 | 0.75 | 400 | 0.0225 | 0.0009 |
| 143 | 2.80 | 0.75 | 500 | 0.0215 | 0.002 |
| 144 | 2.80 | 0.75 | 600 | 0.02 | 0.0035 |
| 145 | 0 | 1 | 100 | 0.6565 | 0.0008 |
| 146 | 0 | 1 | 200 | 0.7085 | 0.117 |
| 147 | 0 | 1 | 300 | 0.7002 | 0.1569 |
| 148 | 0 | 1 | 400 | 0.6802 | 0.1769 |
| 149 | 0 | 1 | 500 | 0.6583 | 0.1988 |
| 150 | 0 | 1 | 600 | 0.6396 | 0.2176 |
| 151 | 0.40 | 1 | 100 | 0.2077 | 0.0017 |
| 152 | 0.40 | 1 | 200 | 0.498 | 0.0375 |
| 153 | 0.40 | 1 | 300 | 0.5402 | 0.0616 |
| 154 | 0.40 | 1 | 400 | 0.5187 | 0.0909 |
| 155 | 0.40 | 1 | 500 | 0.4927 | 0.1183 |
| 156 | 0.40 | 1 | 600 | 0.4703 | 0.1409 |
| 157 | 0.80 | 1 | 100 | 0.1109 | 0.0001 |
| 158 | 0.80 | 1 | 200 | 0.3582 | 0.0046 |
| 159 | 0.80 | 1 | 300 | 0.4075 | 0.0218 |
| 160 | 0.80 | 1 | 400 | 0.3914 | 0.0462 |
| 161 | 0.80 | 1 | 500 | 0.3683 | 0.0707 |
| 162 | 0.80 | 1 | 600 | 0.3474 | 0.092 |
| 163 | 1.20 | 1 | 100 | 0.0722 | 0 |
| 164 | 1.20 | 1 | 200 | 0.2522 | 0.0015 |
| 165 | 1.20 | 1 | 300 | 0.2955 | 0.0091 |
| 166 | 1.20 | 1 | 400 | 0.2871 | 0.024 |
| 167 | 1.20 | 1 | 500 | 0.2701 | 0.0422 |
| 168 | 1.20 | 1 | 600 | 0.253 | 0.0595 |
| 169 | 1.60 | 1 | 100 | 0.0497 | 0 |
| 170 | 1.60 | 1 | 200 | 0.1743 | 0.0007 |
| 171 | 1.60 | 1 | 300 | 0.2052 | 0.0043 |
| 172 | 1.60 | 1 | 400 | 0.2013 | 0.0127 |
| 173 | 1.60 | 1 | 500 | 0.1901 | 0.0246 |
| 174 | 1.60 | 1 | 600 | 0.1776 | 0.0374 |
| 175 | 2.00 | 1 | 100 | 0.0337 | 0 |
| 176 | 2.00 | 1 | 200 | 0.1137 | 0.0003 |
| 177 | 2.00 | 1 | 300 | 0.1324 | 0.0021 |
| 178 | 2.00 | 1 | 400 | 0.1306 | 0.0065 |
| 179 | 2.00 | 1 | 500 | 0.124 | 0.0136 |
| 180 | 2.00 | 1 | 600 | 0.1158 | 0.0219 |
| 181 | 2.40 | 1 | 100 | 0.0206 | 0 |
| 182 | 2.40 | 1 | 200 | 0.0639 | 0.0001 |
| 183 | 2.40 | 1 | 300 | 0.0725 | 0.0009 |
| 184 | 2.40 | 1 | 400 | 0.0717 | 0.003 |
| 185 | 2.40 | 1 | 500 | 0.0684 | 0.0065 |
| 186 | 2.40 | 1 | 600 | 0.0639 | 0.0109 |
| 187 | 2.80 | 1 | 100 | 0.0082 | 0 |
| 188 | 2.80 | 1 | 200 | 0.0206 | 0 |
| 189 | 2.80 | 1 | 300 | 0.0223 | 0.0002 |
| 190 | 2.80 | 1 | 400 | 0.022 | 0.0008 |
| 191 | 2.80 | 1 | 500 | 0.0211 | 0.0017 |
| 192 | 2.80 | 1 | 600 | 0.0197 | 0.0031 |
| 193 | 0 | 1.25 | 100 | 0.6712 | 0.0007 |
| 194 | 0 | 1.25 | 200 | 0.7246 | 0.0693 |
| 195 | 0 | 1.25 | 300 | 0.7032 | 0.0968 |
| 196 | 0 | 1.25 | 400 | 0.673 | 0.127 |
| 197 | 0 | 1.25 | 500 | 0.6442 | 0.1558 |
| 198 | 0 | 1.25 | 600 | 0.6206 | 0.1794 |
| 199 | 0.40 | 1.25 | 100 | 0.2274 | 0.0004 |
| 200 | 0.40 | 1.25 | 200 | 0.4921 | 0.0103 |
| 201 | 0.40 | 1.25 | 300 | 0.5321 | 0.0355 |
| 202 | 0.40 | 1.25 | 400 | 0.5099 | 0.0657 |
| 203 | 0.40 | 1.25 | 500 | 0.483 | 0.0942 |
| 204 | 0.40 | 1.25 | 600 | 0.4593 | 0.118 |
| 205 | 0.80 | 1.25 | 100 | 0.1076 | 0.0001 |
| 206 | 0.80 | 1.25 | 200 | 0.3442 | 0.0026 |
| 207 | 0.80 | 1.25 | 300 | 0.3946 | 0.0143 |
| 208 | 0.80 | 1.25 | 400 | 0.382 | 0.0348 |
| 209 | 0.80 | 1.25 | 500 | 0.3607 | 0.0575 |
| 210 | 0.80 | 1.25 | 600 | 0.3404 | 0.0781 |
| 211 | 1.20 | 1.25 | 100 | 0.0706 | 0 |
| 212 | 1.20 | 1.25 | 200 | 0.2433 | 0.0011 |
| 213 | 1.20 | 1.25 | 300 | 0.2853 | 0.0068 |
| 214 | 1.20 | 1.25 | 400 | 0.2793 | 0.019 |
| 215 | 1.20 | 1.25 | 500 | 0.2643 | 0.035 |
| 216 | 1.20 | 1.25 | 600 | 0.2484 | 0.0511 |
| 217 | 1.60 | 1.25 | 100 | 0.0488 | 0 |
| 218 | 1.60 | 1.25 | 200 | 0.1688 | 0.0005 |
| 219 | 1.60 | 1.25 | 300 | 0.1985 | 0.0035 |
| 220 | 1.60 | 1.25 | 400 | 0.1957 | 0.0104 |
| 221 | 1.60 | 1.25 | 500 | 0.186 | 0.0208 |
| 222 | 1.60 | 1.25 | 600 | 0.1746 | 0.0324 |
| 223 | 2.00 | 1.25 | 100 | 0.0331 | 0 |
| 224 | 2.00 | 1.25 | 200 | 0.1105 | 0.0003 |
| 225 | 2.00 | 1.25 | 300 | 0.1284 | 0.0018 |
| 226 | 2.00 | 1.25 | 400 | 0.127 | 0.0055 |
| 227 | 2.00 | 1.25 | 500 | 0.1213 | 0.0117 |
| 228 | 2.00 | 1.25 | 600 | 0.1139 | 0.0192 |
| 229 | 2.40 | 1.25 | 100 | 0.0203 | 0 |
| 230 | 2.40 | 1.25 | 200 | 0.0622 | 0.0001 |
| 231 | 2.40 | 1.25 | 300 | 0.0705 | 0.0008 |
| 232 | 2.40 | 1.25 | 400 | 0.0698 | 0.0026 |
| 233 | 2.40 | 1.25 | 500 | 0.0669 | 0.0056 |
| 234 | 2.40 | 1.25 | 600 | 0.0629 | 0.0097 |
| 235 | 2.80 | 1.25 | 100 | 0.0081 | 0 |
| 236 | 2.80 | 1.25 | 200 | 0.0201 | 0 |
| 237 | 2.80 | 1.25 | 300 | 0.0217 | 0.0002 |
| 238 | 2.80 | 1.25 | 400 | 0.0215 | 0.0007 |
| 239 | 2.80 | 1.25 | 500 | 0.0206 | 0.0015 |
| 240 | 2.80 | 1.25 | 600 | 0.0194 | 0.0027 |
| 241 | 0 | 1.5 | 100 | 0.6758 | 0.0006 |
| 242 | 0 | 1.5 | 200 | 0.7237 | 0.026 |
| 243 | 0 | 1.5 | 300 | 0.6929 | 0.0571 |
| 244 | 0 | 1.5 | 400 | 0.658 | 0.092 |
| 245 | 0 | 1.5 | 500 | 0.6261 | 0.1239 |
| 246 | 0 | 1.5 | 600 | 0.6002 | 0.1498 |
| 247 | 0.40 | 1.5 | 100 | 0.2169 | 0.0001 |
| 248 | 0.40 | 1.5 | 200 | 0.4715 | 0.0046 |
| 249 | 0.40 | 1.5 | 300 | 0.5154 | 0.0223 |
| 250 | 0.40 | 1.5 | 400 | 0.4963 | 0.049 |
| 251 | 0.40 | 1.5 | 500 | 0.4703 | 0.0764 |
| 252 | 0.40 | 1.5 | 600 | 0.4469 | 0.1 |
| 253 | 0.80 | 1.5 | 100 | 0.1046 | 0 |
| 254 | 0.80 | 1.5 | 200 | 0.3306 | 0.0017 |
| 255 | 0.80 | 1.5 | 300 | 0.3802 | 0.0102 |
| 256 | 0.80 | 1.5 | 400 | 0.3708 | 0.0271 |
| 257 | 0.80 | 1.5 | 500 | 0.3516 | 0.0475 |
| 258 | 0.80 | 1.5 | 600 | 0.3325 | 0.067 |
| 259 | 1.20 | 1.5 | 100 | 0.069 | 0 |
| 260 | 1.20 | 1.5 | 200 | 0.235 | 0.0008 |
| 261 | 1.20 | 1.5 | 300 | 0.2753 | 0.0053 |
| 262 | 1.20 | 1.5 | 400 | 0.2709 | 0.0154 |
| 263 | 1.20 | 1.5 | 500 | 0.2578 | 0.0295 |
| 264 | 1.20 | 1.5 | 600 | 0.2432 | 0.0444 |
| 265 | 1.60 | 1.5 | 100 | 0.0479 | 0 |
| 266 | 1.60 | 1.5 | 200 | 0.1637 | 0.0004 |
| 267 | 1.60 | 1.5 | 300 | 0.192 | 0.0028 |
| 268 | 1.60 | 1.5 | 400 | 0.19 | 0.0087 |
| 269 | 1.60 | 1.5 | 500 | 0.1816 | 0.0179 |
| 270 | 1.60 | 1.5 | 600 | 0.1712 | 0.0284 |
| 271 | 2.00 | 1.5 | 100 | 0.0326 | 0 |
| 272 | 2.00 | 1.5 | 200 | 0.1074 | 0.0002 |
| 273 | 2.00 | 1.5 | 300 | 0.1245 | 0.0015 |
| 274 | 2.00 | 1.5 | 400 | 0.1235 | 0.0048 |
| 275 | 2.00 | 1.5 | 500 | 0.1185 | 0.0102 |
| 276 | 2.00 | 1.5 | 600 | 0.1118 | 0.017 |
| 277 | 2.40 | 1.5 | 100 | 0.02 | 0 |
| 278 | 2.40 | 1.5 | 200 | 0.0606 | 0.0001 |
| 279 | 2.40 | 1.5 | 300 | 0.0685 | 0.0007 |
| 280 | 2.40 | 1.5 | 400 | 0.068 | 0.0022 |
| 281 | 2.40 | 1.5 | 500 | 0.0654 | 0.005 |
| 282 | 2.40 | 1.5 | 600 | 0.0618 | 0.0086 |
| 283 | 2.80 | 1.5 | 100 | 0.008 | 0 |
| 284 | 2.80 | 1.5 | 200 | 0.0196 | 0 |
| 285 | 2.80 | 1.5 | 300 | 0.0212 | 0.0002 |
| 286 | 2.80 | 1.5 | 400 | 0.0209 | 0.0006 |
| 287 | 2.80 | 1.5 | 500 | 0.0202 | 0.0014 |
| 288 | 2.80 | 1.5 | 600 | 0.0191 | 0.0025 |
| 289 | 0 | 1.75 | 100 | 0.6705 | 0.0004 |
| 290 | 0 | 1.75 | 200 | 0.6962 | 0.0097 |
| 291 | 0 | 1.75 | 300 | 0.6709 | 0.035 |
| 292 | 0 | 1.75 | 400 | 0.6376 | 0.0683 |
| 293 | 0 | 1.75 | 500 | 0.6058 | 0.1001 |
| 294 | 0 | 1.75 | 600 | 0.5792 | 0.1266 |
| 295 | 0.40 | 1.75 | 100 | 0.2071 | 0.0001 |
| 296 | 0.40 | 1.75 | 200 | 0.4504 | 0.0028 |
| 297 | 0.40 | 1.75 | 300 | 0.4955 | 0.0154 |
| 298 | 0.40 | 1.75 | 400 | 0.4802 | 0.0378 |
| 299 | 0.40 | 1.75 | 500 | 0.4563 | 0.063 |
| 300 | 0.40 | 1.75 | 600 | 0.4339 | 0.0857 |
| 301 | 0.80 | 1.75 | 100 | 0.1018 | 0 |
| 302 | 0.80 | 1.75 | 200 | 0.318 | 0.0013 |
| 303 | 0.80 | 1.75 | 300 | 0.3659 | 0.0078 |
| 304 | 0.80 | 1.75 | 400 | 0.3588 | 0.0218 |
| 305 | 0.80 | 1.75 | 500 | 0.3418 | 0.04 |
| 306 | 0.80 | 1.75 | 600 | 0.324 | 0.0581 |
| 307 | 1.20 | 1.75 | 100 | 0.0676 | 0 |
| 308 | 1.20 | 1.75 | 200 | 0.2272 | 0.0007 |
| 309 | 1.20 | 1.75 | 300 | 0.2657 | 0.0043 |
| 310 | 1.20 | 1.75 | 400 | 0.2626 | 0.0128 |
| 311 | 1.20 | 1.75 | 500 | 0.251 | 0.0253 |
| 312 | 1.20 | 1.75 | 600 | 0.2376 | 0.0389 |
| 313 | 1.60 | 1.75 | 100 | 0.047 | 0 |
| 314 | 1.60 | 1.75 | 200 | 0.1589 | 0.0004 |
| 315 | 1.60 | 1.75 | 300 | 0.1858 | 0.0024 |
| 316 | 1.60 | 1.75 | 400 | 0.1845 | 0.0075 |
| 317 | 1.60 | 1.75 | 500 | 0.177 | 0.0156 |
| 318 | 1.60 | 1.75 | 600 | 0.1676 | 0.0252 |
| 319 | 2.00 | 1.75 | 100 | 0.0321 | 0 |
| 320 | 2.00 | 1.75 | 200 | 0.1045 | 0.0002 |
| 321 | 2.00 | 1.75 | 300 | 0.1208 | 0.0013 |
| 322 | 2.00 | 1.75 | 400 | 0.1202 | 0.0041 |
| 323 | 2.00 | 1.75 | 500 | 0.1157 | 0.009 |
| 324 | 2.00 | 1.75 | 600 | 0.1096 | 0.0152 |
| 325 | 2.40 | 1.75 | 100 | 0.0197 | 0 |
| 326 | 2.40 | 1.75 | 200 | 0.0591 | 0.0001 |
| 327 | 2.40 | 1.75 | 300 | 0.0667 | 0.0006 |
| 328 | 2.40 | 1.75 | 400 | 0.0662 | 0.002 |
| 329 | 2.40 | 1.75 | 500 | 0.064 | 0.0044 |
| 330 | 2.40 | 1.75 | 600 | 0.0607 | 0.0078 |
| 331 | 2.80 | 1.75 | 100 | 0.0079 | 0 |
| 332 | 2.80 | 1.75 | 200 | 0.0191 | 0 |
| 333 | 2.80 | 1.75 | 300 | 0.0206 | 0.0002 |
| 334 | 2.80 | 1.75 | 400 | 0.0204 | 0.0005 |
| 335 | 2.80 | 1.75 | 500 | 0.0198 | 0.0012 |
| 336 | 2.80 | 1.75 | 600 | 0.0188 | 0.0022 |
| 337 | 0 | 2 | 100 | 0.6533 | 0.0003 |
| 338 | 0 | 2 | 200 | 0.6615 | 0.0052 |
| 339 | 0 | 2 | 300 | 0.6434 | 0.0233 |
| 340 | 0 | 2 | 400 | 0.6145 | 0.0521 |
| 341 | 0 | 2 | 500 | 0.5844 | 0.0822 |
| 342 | 0 | 2 | 600 | 0.5584 | 0.1083 |
| 343 | 0.40 | 2 | 100 | 0.1983 | 0.0001 |
| 344 | 0.40 | 2 | 200 | 0.4307 | 0.002 |
| 345 | 0.40 | 2 | 300 | 0.4753 | 0.0114 |
| 346 | 0.40 | 2 | 400 | 0.4634 | 0.0301 |
| 347 | 0.40 | 2 | 500 | 0.4418 | 0.0528 |
| 348 | 0.40 | 2 | 600 | 0.4208 | 0.0743 |
| 349 | 0.80 | 2 | 100 | 0.0993 | 0 |
| 350 | 0.80 | 2 | 200 | 0.3062 | 0.001 |
| 351 | 0.80 | 2 | 300 | 0.3521 | 0.0062 |
| 352 | 0.80 | 2 | 400 | 0.3469 | 0.0179 |
| 353 | 0.80 | 2 | 500 | 0.3318 | 0.0341 |
| 354 | 0.80 | 2 | 600 | 0.3153 | 0.0509 |
| 355 | 1.20 | 2 | 100 | 0.0662 | 0 |
| 356 | 1.20 | 2 | 200 | 0.2199 | 0.0005 |
| 357 | 1.20 | 2 | 300 | 0.2566 | 0.0036 |
| 358 | 1.20 | 2 | 400 | 0.2543 | 0.0108 |
| 359 | 1.20 | 2 | 500 | 0.2442 | 0.0219 |
| 360 | 1.20 | 2 | 600 | 0.2319 | 0.0344 |
| 361 | 1.60 | 2 | 100 | 0.0462 | 0 |
| 362 | 1.60 | 2 | 200 | 0.1543 | 0.0003 |
| 363 | 1.60 | 2 | 300 | 0.18 | 0.002 |
| 364 | 1.60 | 2 | 400 | 0.1791 | 0.0065 |
| 365 | 1.60 | 2 | 500 | 0.1725 | 0.0137 |
| 366 | 1.60 | 2 | 600 | 0.1639 | 0.0224 |
| 367 | 2.00 | 2 | 100 | 0.0316 | 0 |
| 368 | 2.00 | 2 | 200 | 0.1018 | 0.0002 |
| 369 | 2.00 | 2 | 300 | 0.1174 | 0.0011 |
| 370 | 2.00 | 2 | 400 | 0.1169 | 0.0037 |
| 371 | 2.00 | 2 | 500 | 0.1129 | 0.008 |
| 372 | 2.00 | 2 | 600 | 0.1074 | 0.0136 |
| 373 | 2.40 | 2 | 100 | 0.0194 | 0 |
| 374 | 2.40 | 2 | 200 | 0.0576 | 0.0001 |
| 375 | 2.40 | 2 | 300 | 0.0649 | 0.0005 |
| 376 | 2.40 | 2 | 400 | 0.0646 | 0.0018 |
| 377 | 2.40 | 2 | 500 | 0.0625 | 0.004 |
| 378 | 2.40 | 2 | 600 | 0.0595 | 0.007 |
| 379 | 2.80 | 2 | 100 | 0.0077 | 0 |
| 380 | 2.80 | 2 | 200 | 0.0187 | 0 |
| 381 | 2.80 | 2 | 300 | 0.0201 | 0.0001 |
| 382 | 2.80 | 2 | 400 | 0.0199 | 0.0005 |
| 383 | 2.80 | 2 | 500 | 0.0193 | 0.0011 |
| 384 | 2.80 | 2 | 600 | 0.0184 | 0.002 |
| 385 | 0 | 2.25 | 100 | 0.6533 | 0.0003 |
| 386 | 0 | 2.25 | 200 | 0.6615 | 0.0052 |
| 387 | 0 | 2.25 | 300 | 0.6434 | 0.0233 |
| 388 | 0 | 2.25 | 400 | 0.6145 | 0.0521 |
| 389 | 0 | 2.25 | 500 | 0.5844 | 0.0822 |
| 390 | 0 | 2.25 | 600 | 0.5584 | 0.1083 |
| 391 | 0.40 | 2.25 | 100 | 0.1983 | 0.0001 |
| 392 | 0.40 | 2.25 | 200 | 0.4307 | 0.002 |
| 393 | 0.40 | 2.25 | 300 | 0.4753 | 0.0114 |
| 394 | 0.40 | 2.25 | 400 | 0.4634 | 0.0301 |
| 395 | 0.40 | 2.25 | 500 | 0.4418 | 0.0528 |
| 396 | 0.40 | 2.25 | 600 | 0.4208 | 0.0743 |
| 397 | 0.80 | 2.25 | 100 | 0.0993 | 0 |
| 398 | 0.80 | 2.25 | 200 | 0.3062 | 0.001 |
| 399 | 0.80 | 2.25 | 300 | 0.3521 | 0.0062 |
| 400 | 0.80 | 2.25 | 400 | 0.3469 | 0.0179 |
| 401 | 0.80 | 2.25 | 500 | 0.3318 | 0.0341 |
| 402 | 0.80 | 2.25 | 600 | 0.3153 | 0.0509 |
| 403 | 1.20 | 2.25 | 100 | 0.0662 | 0 |
| 404 | 1.20 | 2.25 | 200 | 0.2199 | 0.0005 |
| 405 | 1.20 | 2.25 | 300 | 0.2566 | 0.0036 |
| 406 | 1.20 | 2.25 | 400 | 0.2543 | 0.0108 |
| 407 | 1.20 | 2.25 | 500 | 0.2442 | 0.0219 |
| 408 | 1.20 | 2.25 | 600 | 0.2319 | 0.0344 |
| 409 | 1.60 | 2.25 | 100 | 0.0462 | 0 |
| 410 | 1.60 | 2.25 | 200 | 0.1543 | 0.0003 |
| 411 | 1.60 | 2.25 | 300 | 0.18 | 0.002 |
| 412 | 1.60 | 2.25 | 400 | 0.1791 | 0.0065 |
| 413 | 1.60 | 2.25 | 500 | 0.1725 | 0.0137 |
| 414 | 1.60 | 2.25 | 600 | 0.1639 | 0.0224 |
| 415 | 2.00 | 2.25 | 100 | 0.0316 | 0 |
| 416 | 2.00 | 2.25 | 200 | 0.1018 | 0.0002 |
| 417 | 2.00 | 2.25 | 300 | 0.1174 | 0.0011 |
| 418 | 2.00 | 2.25 | 400 | 0.1169 | 0.0037 |
| 419 | 2.00 | 2.25 | 500 | 0.1129 | 0.008 |
| 420 | 2.00 | 2.25 | 600 | 0.1074 | 0.0136 |
| 421 | 2.40 | 2.25 | 100 | 0.0194 | 0 |
| 422 | 2.40 | 2.25 | 200 | 0.0576 | 0.0001 |
| 423 | 2.40 | 2.25 | 300 | 0.0649 | 0.0005 |
| 424 | 2.40 | 2.25 | 400 | 0.0646 | 0.0018 |
| 425 | 2.40 | 2.25 | 500 | 0.0625 | 0.004 |
| 426 | 2.40 | 2.25 | 600 | 0.0595 | 0.007 |
| 427 | 2.80 | 2.25 | 100 | 0.0077 | 0 |
| 428 | 2.80 | 2.25 | 200 | 0.0187 | 0 |
| 429 | 2.80 | 2.25 | 300 | 0.0201 | 0.0001 |
| 430 | 2.80 | 2.25 | 400 | 0.0199 | 0.0005 |
| 431 | 2.80 | 2.25 | 500 | 0.0193 | 0.0011 |
| 432 | 2.80 | 2.25 | 600 | 0.0184 | 0.002 |
| 433 | 0 | 2.5 | 100 | 0.5982 | 0.0002 |
| 434 | 0 | 2.5 | 200 | 0.5975 | 0.0025 |
| 435 | 0 | 2.5 | 300 | 0.5874 | 0.0126 |
| 436 | 0 | 2.5 | 400 | 0.5669 | 0.0331 |
| 437 | 0 | 2.5 | 500 | 0.542 | 0.058 |
| 438 | 0 | 2.5 | 600 | 0.5185 | 0.0815 |
| 439 | 0.40 | 2.5 | 100 | 0.1833 | 0 |
| 440 | 0.40 | 2.5 | 200 | 0.3958 | 0.0012 |
| 441 | 0.40 | 2.5 | 300 | 0.4375 | 0.0071 |
| 442 | 0.40 | 2.5 | 400 | 0.4302 | 0.0204 |
| 443 | 0.40 | 2.5 | 500 | 0.4131 | 0.0385 |
| 444 | 0.40 | 2.5 | 600 | 0.3947 | 0.0572 |
| 445 | 0.80 | 2.5 | 100 | 0.0946 | 0 |
| 446 | 0.80 | 2.5 | 200 | 0.2852 | 0.0007 |
| 447 | 0.80 | 2.5 | 300 | 0.3269 | 0.0043 |
| 448 | 0.80 | 2.5 | 400 | 0.324 | 0.0128 |
| 449 | 0.80 | 2.5 | 500 | 0.3121 | 0.0257 |
| 450 | 0.80 | 2.5 | 600 | 0.2981 | 0.04 |
| 451 | 1.20 | 2.5 | 100 | 0.0637 | 0 |
| 452 | 1.20 | 2.5 | 200 | 0.2066 | 0.0004 |
| 453 | 1.20 | 2.5 | 300 | 0.2399 | 0.0026 |
| 454 | 1.20 | 2.5 | 400 | 0.2388 | 0.0081 |
| 455 | 1.20 | 2.5 | 500 | 0.2308 | 0.0169 |
| 456 | 1.20 | 2.5 | 600 | 0.2205 | 0.0275 |
| 457 | 1.60 | 2.5 | 100 | 0.0447 | 0 |
| 458 | 1.60 | 2.5 | 200 | 0.146 | 0.0002 |
| 459 | 1.60 | 2.5 | 300 | 0.1694 | 0.0016 |
| 460 | 1.60 | 2.5 | 400 | 0.169 | 0.005 |
| 461 | 1.60 | 2.5 | 500 | 0.1637 | 0.0108 |
| 462 | 1.60 | 2.5 | 600 | 0.1565 | 0.0182 |
| 463 | 2.00 | 2.5 | 100 | 0.0307 | 0 |
| 464 | 2.00 | 2.5 | 200 | 0.0968 | 0.0001 |
| 465 | 2.00 | 2.5 | 300 | 0.111 | 0.0009 |
| 466 | 2.00 | 2.5 | 400 | 0.1108 | 0.0029 |
| 467 | 2.00 | 2.5 | 500 | 0.1076 | 0.0065 |
| 468 | 2.00 | 2.5 | 600 | 0.1029 | 0.0112 |
| 469 | 2.40 | 2.5 | 100 | 0.0189 | 0 |
| 470 | 2.40 | 2.5 | 200 | 0.055 | 0.0001 |
| 471 | 2.40 | 2.5 | 300 | 0.0616 | 0.0004 |
| 472 | 2.40 | 2.5 | 400 | 0.0614 | 0.0014 |
| 473 | 2.40 | 2.5 | 500 | 0.0598 | 0.0033 |
| 474 | 2.40 | 2.5 | 600 | 0.0572 | 0.0058 |
| 475 | 2.80 | 2.5 | 100 | 0.0075 | 0 |
| 476 | 2.80 | 2.5 | 200 | 0.0179 | 0 |
| 477 | 2.80 | 2.5 | 300 | 0.0192 | 0.0001 |
| 478 | 2.80 | 2.5 | 400 | 0.019 | 0.0004 |
| 479 | 2.80 | 2.5 | 500 | 0.0185 | 0.0009 |
| 480 | 2.80 | 2.5 | 600 | 0.0178 | 0.0017 |
| 481 | 0 | 3 | 100 | 0.545 | 0.0001 |
| 482 | 0 | 3 | 200 | 0.5439 | 0.0015 |
| 483 | 0 | 3 | 300 | 0.5373 | 0.0081 |
| 484 | 0 | 3 | 400 | 0.5226 | 0.0229 |
| 485 | 0 | 3 | 500 | 0.5025 | 0.043 |
| 486 | 0 | 3 | 600 | 0.4821 | 0.0634 |
| 487 | 0.40 | 3 | 100 | 0.1709 | 0 |
| 488 | 0.40 | 3 | 200 | 0.3662 | 0.0008 |
| 489 | 0.40 | 3 | 300 | 0.4044 | 0.005 |
| 490 | 0.40 | 3 | 400 | 0.3997 | 0.0148 |
| 491 | 0.40 | 3 | 500 | 0.3861 | 0.0294 |
| 492 | 0.40 | 3 | 600 | 0.3704 | 0.0454 |
| 493 | 0.80 | 3 | 100 | 0.0906 | 0 |
| 494 | 0.80 | 3 | 200 | 0.2669 | 0.0005 |
| 495 | 0.80 | 3 | 300 | 0.3046 | 0.0031 |
| 496 | 0.80 | 3 | 400 | 0.3031 | 0.0097 |
| 497 | 0.80 | 3 | 500 | 0.2936 | 0.0201 |
| 498 | 0.80 | 3 | 600 | 0.2817 | 0.0322 |
| 499 | 1.20 | 3 | 100 | 0.0615 | 0 |
| 500 | 1.20 | 3 | 200 | 0.1949 | 0.0003 |
| 501 | 1.20 | 3 | 300 | 0.2251 | 0.002 |
| 502 | 1.20 | 3 | 400 | 0.2247 | 0.0063 |
| 503 | 1.20 | 3 | 500 | 0.2183 | 0.0135 |
| 504 | 1.20 | 3 | 600 | 0.2095 | 0.0225 |
| 505 | 1.60 | 3 | 100 | 0.0434 | 0 |
| 506 | 1.60 | 3 | 200 | 0.1385 | 0.0002 |
| 507 | 1.60 | 3 | 300 | 0.1598 | 0.0012 |
| 508 | 1.60 | 3 | 400 | 0.1599 | 0.004 |
| 509 | 1.60 | 3 | 500 | 0.1555 | 0.0088 |
| 510 | 1.60 | 3 | 600 | 0.1494 | 0.0151 |
| 511 | 2.00 | 3 | 100 | 0.0298 | 0 |
| 512 | 2.00 | 3 | 200 | 0.0922 | 0.0001 |
| 513 | 2.00 | 3 | 300 | 0.1052 | 0.0007 |
| 514 | 2.00 | 3 | 400 | 0.1052 | 0.0024 |
| 515 | 2.00 | 3 | 500 | 0.1025 | 0.0053 |
| 516 | 2.00 | 3 | 600 | 0.0986 | 0.0094 |
| 517 | 2.40 | 3 | 100 | 0.0184 | 0 |
| 518 | 2.40 | 3 | 200 | 0.0525 | 0.0001 |
| 519 | 2.40 | 3 | 300 | 0.0586 | 0.0004 |
| 520 | 2.40 | 3 | 400 | 0.0585 | 0.0012 |
| 521 | 2.40 | 3 | 500 | 0.0571 | 0.0028 |
| 522 | 2.40 | 3 | 600 | 0.055 | 0.0049 |
| 523 | 2.80 | 3 | 100 | 0.0074 | 0 |
| 524 | 2.80 | 3 | 200 | 0.0171 | 0 |
| 525 | 2.80 | 3 | 300 | 0.0183 | 0.0001 |
| 526 | 2.80 | 3 | 400 | 0.0182 | 0.0003 |
| 527 | 2.80 | 3 | 500 | 0.0178 | 0.0008 |
| 528 | 2.80 | 3 | 600 | 0.0171 | 0.0015 |
| 529 | 0 | 3.5 | 100 | 0.4998 | 0.0001 |
| 530 | 0 | 3.5 | 200 | 0.499 | 0.001 |
| 531 | 0 | 3.5 | 300 | 0.4943 | 0.0057 |
| 532 | 0 | 3.5 | 400 | 0.4832 | 0.0168 |
| 533 | 0 | 3.5 | 500 | 0.4669 | 0.0331 |
| 534 | 0 | 3.5 | 600 | 0.4493 | 0.0507 |
| 535 | 0.40 | 3.5 | 100 | 0.1604 | 0 |
| 536 | 0.40 | 3.5 | 200 | 0.3411 | 0.0006 |
| 537 | 0.40 | 3.5 | 300 | 0.3757 | 0.0037 |
| 538 | 0.40 | 3.5 | 400 | 0.3726 | 0.0113 |
| 539 | 0.40 | 3.5 | 500 | 0.3616 | 0.0232 |
| 540 | 0.40 | 3.5 | 600 | 0.3481 | 0.0369 |
| 541 | 0.80 | 3.5 | 100 | 0.0869 | 0 |
| 542 | 0.80 | 3.5 | 200 | 0.2509 | 0.0004 |
| 543 | 0.80 | 3.5 | 300 | 0.2851 | 0.0024 |
| 544 | 0.80 | 3.5 | 400 | 0.2844 | 0.0076 |
| 545 | 0.80 | 3.5 | 500 | 0.2766 | 0.0162 |
| 546 | 0.80 | 3.5 | 600 | 0.2665 | 0.0266 |
| 547 | 1.20 | 3.5 | 100 | 0.0595 | 0 |
| 548 | 1.20 | 3.5 | 200 | 0.1845 | 0.0002 |
| 549 | 1.20 | 3.5 | 300 | 0.212 | 0.0016 |
| 550 | 1.20 | 3.5 | 400 | 0.212 | 0.0051 |
| 551 | 1.20 | 3.5 | 500 | 0.2067 | 0.0111 |
| 552 | 1.20 | 3.5 | 600 | 0.1992 | 0.0187 |
| 553 | 1.60 | 3.5 | 100 | 0.0421 | 0 |
| 554 | 1.60 | 3.5 | 200 | 0.1318 | 0.0002 |
| 555 | 1.60 | 3.5 | 300 | 0.1512 | 0.001 |
| 556 | 1.60 | 3.5 | 400 | 0.1515 | 0.0033 |
| 557 | 1.60 | 3.5 | 500 | 0.1479 | 0.0073 |
| 558 | 1.60 | 3.5 | 600 | 0.1426 | 0.0127 |
| 559 | 2.00 | 3.5 | 100 | 0.0291 | 0 |
| 560 | 2.00 | 3.5 | 200 | 0.088 | 0.0001 |
| 561 | 2.00 | 3.5 | 300 | 0.1 | 0.0006 |
| 562 | 2.00 | 3.5 | 400 | 0.1001 | 0.002 |
| 563 | 2.00 | 3.5 | 500 | 0.0979 | 0.0045 |
| 564 | 2.00 | 3.5 | 600 | 0.0944 | 0.008 |
| 565 | 2.40 | 3.5 | 100 | 0.018 | 0 |
| 566 | 2.40 | 3.5 | 200 | 0.0503 | 0 |
| 567 | 2.40 | 3.5 | 300 | 0.0559 | 0.0003 |
| 568 | 2.40 | 3.5 | 400 | 0.0559 | 0.001 |
| 569 | 2.40 | 3.5 | 500 | 0.0547 | 0.0023 |
| 570 | 2.40 | 3.5 | 600 | 0.0528 | 0.0042 |
| 571 | 2.80 | 3.5 | 100 | 0.0072 | 0 |
| 572 | 2.80 | 3.5 | 200 | 0.0164 | 0 |
| 573 | 2.80 | 3.5 | 300 | 0.0175 | 0.0001 |
| 574 | 2.80 | 3.5 | 400 | 0.0174 | 0.0003 |
| 575 | 2.80 | 3.5 | 500 | 0.0171 | 0.0007 |
| 576 | 2.80 | 3.5 | 600 | 0.0165 | 0.0013 |
| 577 | 0 | 4 | 100 | 0.4615 | 0 |
| 578 | 0 | 4 | 200 | 0.4608 | 0.0008 |
| 579 | 0 | 4 | 300 | 0.4572 | 0.0043 |
| 580 | 0 | 4 | 400 | 0.4486 | 0.013 |
| 581 | 0 | 4 | 500 | 0.4353 | 0.0263 |
| 582 | 0 | 4 | 600 | 0.4201 | 0.0414 |
| 583 | 0.40 | 4 | 100 | 0.1515 | 0 |
| 584 | 0.40 | 4 | 200 | 0.319 | 0.0005 |
| 585 | 0.40 | 4 | 300 | 0.3505 | 0.0029 |
| 586 | 0.40 | 4 | 400 | 0.3485 | 0.009 |
| 587 | 0.40 | 4 | 500 | 0.3395 | 0.0188 |
| 588 | 0.40 | 4 | 600 | 0.3279 | 0.0305 |
| 589 | 0.80 | 4 | 100 | 0.0837 | 0 |
| 590 | 0.80 | 4 | 200 | 0.2368 | 0.0003 |
| 591 | 0.80 | 4 | 300 | 0.2679 | 0.0019 |
| 592 | 0.80 | 4 | 400 | 0.2676 | 0.0062 |
| 593 | 0.80 | 4 | 500 | 0.2612 | 0.0133 |
| 594 | 0.80 | 4 | 600 | 0.2525 | 0.0223 |
| 595 | 1.20 | 4 | 100 | 0.0576 | 0 |
| 596 | 1.20 | 4 | 200 | 0.1752 | 0.0002 |
| 597 | 1.20 | 4 | 300 | 0.2003 | 0.0013 |
| 598 | 1.20 | 4 | 400 | 0.2006 | 0.0042 |
| 599 | 1.20 | 4 | 500 | 0.1961 | 0.0093 |
| 600 | 1.20 | 4 | 600 | 0.1896 | 0.0159 |
| 601 | 1.60 | 4 | 100 | 0.041 | 0 |
| 602 | 1.60 | 4 | 200 | 0.1257 | 0.0001 |
| 603 | 1.60 | 4 | 300 | 0.1435 | 0.0008 |
| 604 | 1.60 | 4 | 400 | 0.1439 | 0.0028 |
| 605 | 1.60 | 4 | 500 | 0.1409 | 0.0062 |
| 606 | 1.60 | 4 | 600 | 0.1363 | 0.0109 |
| 607 | 2.00 | 4 | 100 | 0.0283 | 0 |
| 608 | 2.00 | 4 | 200 | 0.0843 | 0.0001 |
| 609 | 2.00 | 4 | 300 | 0.0952 | 0.0005 |
| 610 | 2.00 | 4 | 400 | 0.0954 | 0.0017 |
| 611 | 2.00 | 4 | 500 | 0.0935 | 0.0038 |
| 612 | 2.00 | 4 | 600 | 0.0906 | 0.0069 |
| 613 | 2.40 | 4 | 100 | 0.0176 | 0 |
| 614 | 2.40 | 4 | 200 | 0.0482 | 0 |
| 615 | 2.40 | 4 | 300 | 0.0534 | 0.0003 |
| 616 | 2.40 | 4 | 400 | 0.0535 | 0.0009 |
| 617 | 2.40 | 4 | 500 | 0.0524 | 0.002 |
| 618 | 2.40 | 4 | 600 | 0.0508 | 0.0037 |
| 619 | 2.80 | 4 | 100 | 0.007 | 0 |
| 620 | 2.80 | 4 | 200 | 0.0157 | 0 |
| 621 | 2.80 | 4 | 300 | 0.0168 | 0.0001 |
| 622 | 2.80 | 4 | 400 | 0.0167 | 0.0003 |
| 623 | 2.80 | 4 | 500 | 0.0164 | 0.0006 |
| 624 | 2.80 | 4 | 600 | 0.0159 | 0.0011 |
